# Bioactive Steroids from the Red Sea Soft Coral *Sinularia polydactyla*

**DOI:** 10.3390/md18120632

**Published:** 2020-12-10

**Authors:** Mohamed A. Tammam, Lucie Rárová, Marie Kvasnicová, Gabriel Gonzalez, Ahmed M. Emam, Aldoushy Mahdy, Miroslav Strnad, Efstathia Ioannou, Vassilios Roussis

**Affiliations:** 1 Section of Pharmacognosy and Chemistry of Natural Products, Department of Pharmacy, National and Kapodistrian University of Athens, Panepistimiopolis Zografou, 15771 Athens, Greece; mtammam@pharm.uoa.gr (M.A.T.); eioannou@pharm.uoa.gr (E.I.); 2 Department of Biochemistry, Faculty of Agriculture, Fayoum University, Fayoum 63514, Egypt; ame01@fayoum.edu.eg; 3 Laboratory of Growth Regulators, Institute of Experimental Botany, The Czech Academy of Sciences, Faculty of Science, Palacký University, Šlechtitelů 27, CZ-78371 Olomouc, Czech Republic; kvasnicova@ueb.cas.cz (M.K.); gonzalez.gabriel@seznam.cz (G.G.); miroslav.strnad@upol.cz (M.S.); 4 Department of Zoology, Faculty of Science, Al-Azhar University (Assiut Branch), Assiut 71524, Egypt; aldoushy@azhar.edu.eg

**Keywords:** *Sinularia polydactyla*, soft coral, steroids, cytotoxic, anti-inflammatory, neuroprotective, androgen receptor

## Abstract

Six new (**1**, **2**, **6**, **8**, **13,** and **20**) and twenty previously isolated (**3**–**5**, **7**, **9**–**12**, **14**–**19**, and **21**–**26**) steroids featuring thirteen different carbocycle motifs were isolated from the organic extract of the soft coral *Sinularia polydactyla* collected from the Hurghada reef in the Red Sea. The structures and the relative configurations of the isolated natural products have been determined based on extensive analysis of their NMR and MS data. The cytotoxic, anti-inflammatory, anti-angiogenic, and neuroprotective activity of compounds **3**–**7**, **9**–**12**, **14**–**20**, and **22**–**26**, as well as their effect on androgen receptor-regulated transcription was evaluated in vitro in human tumor and non-cancerous cells. Steroids **22** and **23** showed significant cytotoxicity in the low micromolar range against the HeLa and MCF7 cancer cell lines, while migration of endothelial cells was inhibited by compounds **11**, **12**, **22,** and **23** at 20 µM. The results of the androgen receptor (AR) reporter assay showed that compound **11** exhibited the strongest inhibition of AR at 10 µM, while it is noteworthy that steroids **10**, **16,** and **20** displayed increased inhibition of AR with decreasing concentrations. Additionally, compounds **11** and **23** showed neuroprotective activity on neuron-like SH-SY5Y cells.

## 1. Introduction

The Red Sea, one of the warmest and most saline marine habitats, is an extension of the Indian Ocean, located between the Arabian Peninsula and Africa. The entire coastal reef complex extends along a 2000-km shoreline and is characterized by a high degree of chemodiversity, including more than 200 soft and 300 hard coral species [1]. Despite the diverse marine life hosted in the Red Sea, marine organisms from this ecosystem have not been thoroughly studied in comparison to other extended coral habitats, such as those encountered in the Great Barrier Reef or the Caribbean Sea [2].

Over the last 50 years, soft corals (Anthozoa, Gorgonacea) have been the subject of extensive chemical investigations which have resulted in the isolation of a large of number secondary metabolites, mainly sesquiterpenes, diterpenes, prostanoids, and highly functionalized steroids [3]. A significant number of these metabolites have exhibited potent biological properties, including cytotoxic, antibacterial, antifungal, anti-inflammatory, and antifouling activity [2]. Among soft corals, species of the genus *Sinularia* have been extensively studied as sources of new bioactive compounds, most often leading to the isolation of diterpenes and steroids with noteworthy levels of bioactivity [2,3].

**Figure 1:**
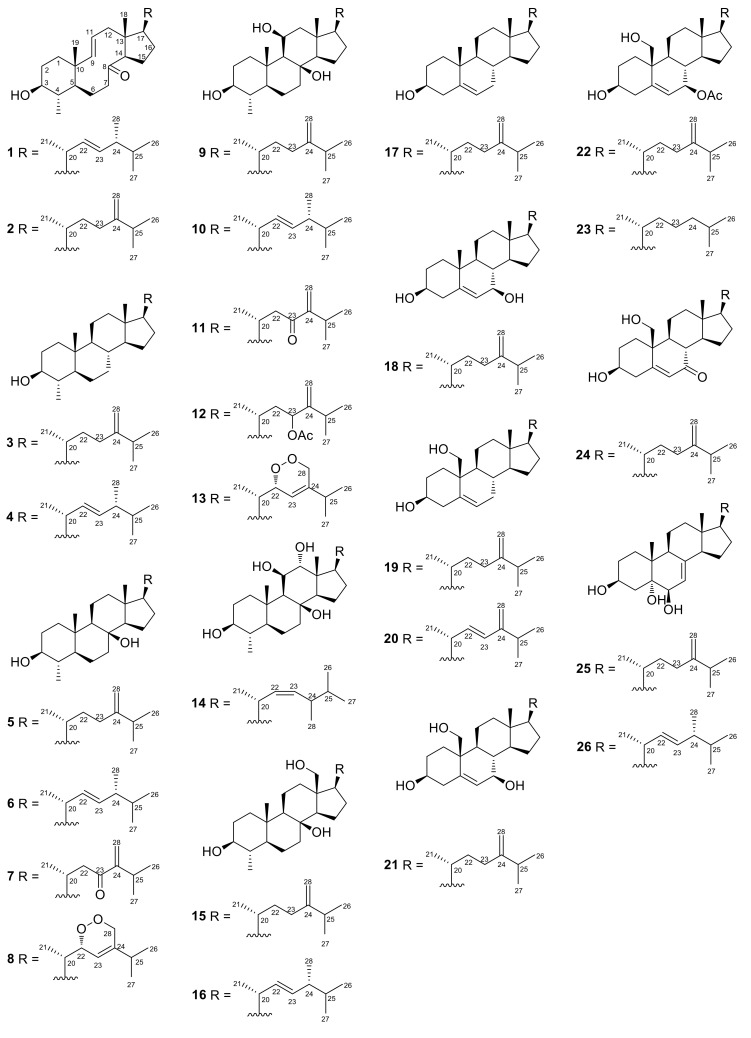
Chemical structures of compounds **1**–**26**.

In the context of our continuous interest for the isolation of bioactive metabolites from marine organisms, we recently had the opportunity to collect specimens of *Sinularia polydactyla* from the coastline of Hurghada in the Red Sea (Egypt) and investigate its chemical profile. Herein we report the isolation, structure elucidation, and evaluation of biological activity of six new (**1**, **2**, **6**, **8**, **13,** and **20**) and twenty previously isolated (**3**–**5**, **7**, **9**–**12**, **14**–**19,** and **21**–**26**) steroids (Figure 1).

## 2. Results and Discussion

### 2.1. Structure Elucidation of the Isolated Metabolites

A series of normal- and reversed-phase chromatographic separations of the organic extract of the soft coral *S. polydactyla* collected from the Egyptian Red Sea coastline at Hurghada allowed for the isolation of compounds **1**–**26**.

**Figure 2:**
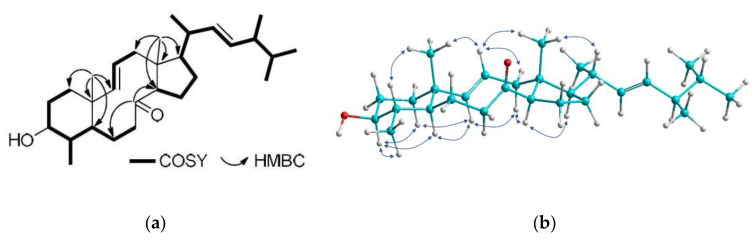
(**a**) COSY and key HMBC correlations and (**b**) key NOESY cross-peaks for compound **1**.

Compound **1**, isolated as a white amorphous solid, possessed the molecular formula C_29_H_48_O_2_, as indicated by the HR-APCIMS and NMR data. The ^13^C NMR and HSQC-DEPT spectra revealed the presence of 29 carbon atoms, corresponding to seven methyls, seven methylenes, twelve methines, and three non-protonated carbon atoms. Among them, evident were one carbonyl resonating at *δ*_C_ 213.1, four olefinic carbons resonating at *δ*_C_ 127.6, 132.6, 135.2, and 142.0 and an oxygenated carbon resonating at *δ*_C_ 76.6. In the ^1^H NMR spectrum evident were two methyls on non-protonated carbons (*δ*_H_ 0.94 and 1.08), five methyls on tertiary carbons (*δ*_H_ 0.80, 0.81, 0.89, 0.99, and 1.04), one oxygenated methine (*δ*_H_ 3.09), and four olefinic methines (*δ*_H_ 5.15, 5.20, 5.28, and 5.41). Since the carbonyl moiety and the two carbon–carbon double bonds accounted for three of the six degrees of unsaturation, the molecular structure of **1** was determined as tricyclic. The spectroscopic features of metabolite **1** (Table 1 and Table 2), in conjunction with the homonuclear and heteronuclear correlations observed in its HSQC, HMBC, and COSY spectra (Figure 2) suggested that compound **1** possessed a 8-oxo-3-hydroxy-4-methyl-8,9-seco steroidal nucleus with a Δ^9,11^ and a C_9_H_17_ unsaturated side chain with a 1,2-disubstituted double bond between C-22 and C-23. Specifically, the correlations observed in the COSY spectrum identified three distinct spin systems, namely (a) a spin system starting from H-1 to H_2_-7, incorporating H_3_-29 which was coupled to H-4, (b) a short spin system from H-9 to H_2_-12 through H-11, and (c) an extended branched spin system from H-14 to H_3_-26, including H_3_-21, H_3_-27, and H_3_-28 which were coupled to H-20, H-25, and H-24, respectively. The HMBC correlations of H_3_-19 with C-1, C-5, C-9, and C-10 concluded the six-membered ring and positioned the first angular methyl on C-10, connecting at the same time spin systems (a) and (b). Additionally, the HMBC correlations of H_3_-18 with C-12, C-13, C-14, and C-17 identified the five-membered ring and fixed the position of the second angular methyl on C-13, connecting spin systems (b) and (c). The HMBC correlations of H_2_-6, H_2_-7, and H-14 with the carbonyl carbon C-8 supported the cleavage of the C-8/C-9 bond, giving rise to the decalin ring and connecting spin systems (a) and (c). The geometry of the two double bonds was determined as *E* in both cases on the basis of the measured coupling constants (*J*_9,11_ = 15.3 Hz and *J*_22,23_ = 15.2 Hz). The enhancements of H-3/H-5, H-3/H_3_-29, H-4/H_3_-19, H-5/H-9, H-9/H-12α, H-9/H-14, H-11/H-12β, H-11/H_3_-18, H-11/H_3_-19, H-14/H-17, and H_3_-18/H-20 observed in the NOESY spectrum (Figure 2) verified the *trans* fusion of the six-membered and the ten-membered rings, as well as the *trans* fusion of the latter with the five-membered ring and determined the relative configuration of the stereogenic centers. The *R* configuration at C-24 was proposed on the basis of the 0.3 ppm difference in the chemical shifts of C-26 and C-27 and the chemical shift of C-28 resonating at 17.6 ppm [4]. On the basis of the above, metabolite **1** was identified as (9*E*,22*E*,24*R*)-3*β*-hydroxy-4*α*,24-dimethyl-8,9-seco-5α-cholesta-9(11),22-dien-8-one.

Compound **2**, isolated in minute amount as a white amorphous solid, displayed an ion peak at *m/z* 429.3725 (HR-APCIMS), corresponding to C_29_H_49_O_2_ and consistent for [M + H]^+^. Compound **2** shared quite similar spectroscopic features with **1**. In particular, all signals attributed to the steroidal nucleus, with the most prominent being the signals of the two angular methyls H_3_-18 and H_3_-19 (*δ*_H_ 1.09 and 0.94, respectively), the methyl at C-4 (*δ*_H_ 1.04), the oxymethine H-3 (*δ*_H_ 3.10), and the two olefinic protons H-9 and H-11 (*δ*_H_ 5.27 and 5.41, respectively), were also evident in the ^1^H NMR spectrum of compound **2** (Table 1). The most significant difference observed was the replacement of the 1,2-disubstituted double bond in the side chain of **1** by a 1,1-disustituted double bond (*δ*_H_ 4.63 and 4.70) in the side chain of **2**. The correlations observed in the COSY spectrum of **2** identified the relevant spin systems; however, metabolite **2** was proven unstable and degraded prior to the acquisition of heteronuclear NMR spectra. Nevertheless, the high structural similarity of **2** with compound **1** renders safe the proposed identification of **2** as (9*E*)-3*β*-hydroxy-4*α*,24-dimethyl-8,9-seco-5α-cholesta-9(11),24(28)-dien-8-one.

**Figure 3:**
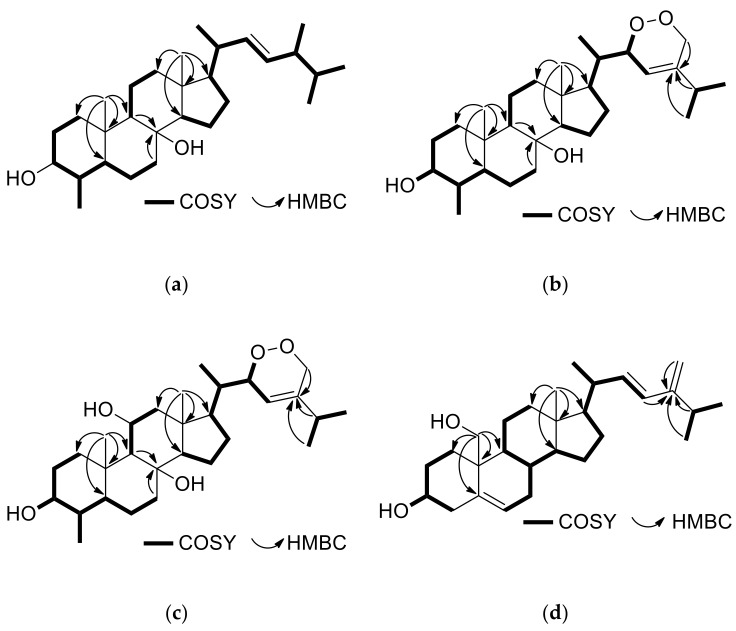
(**a**) COSY and important HMBC correlations for compound **6**. (**b**) COSY and important HMBC correlations for compound **8**. (**c**) COSY and important HMBC correlations for compound **13**. (**d**) COSY and important HMBC correlations for compound **20**.

Compound **6**, isolated as a white amorphous solid, possessed the molecular formula C_29_H_50_O_2_, as suggested by its HR-APCIMS and NMR data. The spectroscopic data of **6** were quite similar to those of the previously reported metabolites **5** and **7** (Table 1 and Table 2). The presence of the two angular methyls at *δ*_H_ 0.92 and 0.97, the doublet methyl at *δ*_H_ 0.95, the oxygenated methine at *δ*_H_ 3.04 and the quaternary oxygenated carbon at *δ*_C_ 73.6, in conjunction with the correlations observed in the HMBC and COSY spectra (Figure 3a), verified the 3,8-dihydroxy-4-methyl steroidal nucleus. The side chain of compound **6** included four doublet methyls (*δ*_H/C_ 0.79/20.2, 0.81/19.9, 0.88/18.0, and 0.95/16.0) and two olefinic methines (*δ*_H/C_ 5.09/135.7 and 5.16/131.8) that was assigned on the basis of the COSY and HMBC correlations. The *E* geometry of the Δ^22^ double bond was supported by the large coupling constant of H-22/H-23 (*J* = 15.3 Hz). The enhancements of H-3/H-5, H-3/H_3_-29, H-4/H_3_-19, H-5/H-9, H-5/H_3_-29, H-9/H-12α, H-9/H-14, H-12β/H_3_-18, and H_3_-18/H-20 observed in the NOESY spectrum verified the *trans* fusion of rings A/B, B/C, and C/D and suggested the axial orientation of the hydroxy group at C-8. The configuration at C-24 was proposed as *R* due to the fact that the difference in the chemical shifts of C-26 and C-27 was 0.3 ppm and that C-28 resonated at 18.0 ppm [4]. Therefore, compound **6** was identified as (22*E*,24*R*)-4*α*,24-dimethyl-5*α*-cholest-22-en-3*β*,8*β-*diol.

Compound **8**, isolated as a white amorphous solid, exhibited an ion peak at *m/z* 459.3475 corresponding to C_29_H_47_O_4_ and consistent with [M − H]^−^. The high degree of similarity of the spectroscopic data of metabolite **8** (Table 1 and Table 2) with those of **5**–**7** indicated the same 3,8-dihydroxy-4-methyl steroidal nucleus, further confirmed by the correlations observed in the HMBC and COSY spectra (Figure 3b). Taking into account that the steroidal nucleus of **8** accounts for four of the six degrees of unsaturation and the presence of one double bond on the side chain, the latter should also contain an additional ring. The ^1^H and ^13^C NMR signals corresponding to the side chain of compound **8** included three doublet methyls (*δ*_H/C_ 0.95/14.9, 1.05/21.0, and 1.05/21.0), one oxygenated methine (*δ*_H/C_ 4.72/79.9), one oxygenated methylene (*δ*_H/C_ 4.20, 4.62/70.6), one olefinic methine (*δ*_H/C_ 5.36/119.4), and one non-protonated olefinic carbon (*δ*_C_ 142.0). The COSY cross-peaks of H-20/H_3_-21, H-20/H-22, H-22/H-23, H-25/H_3_-26, and H-25/H_3_-27, in combination with the HMBC correlations of H_3_-21 with C-17, C-20, and C-22, of H-23, H-25, H_3_-26, and H_3_-27 with C-24 and of H_2_-28 with C-23, C-24, and C-25 verified the side chain. In accordance with the literature, H-22 was assigned to be on the opposite side of H_3_-21, as also suggested by the chemical shift of C-23 which resonated at 119.4 ppm. Instead, when H-22 and H_3_-21 are co-planar, C-23 is shielded, resonating at 115–116 ppm [5]. Thus, metabolite **8** was identified as (23*E*)-22*α*,28-epidioxy-4*α*,24-dimethyl-5*α*-cholest-23-en-3*β*,8*β*-diol.

Compound **13** was isolated as a white amorphous solid. The ion peak at *m/z* 475.3424 observed in its HR-APCIMS was consistent with [M − H]^−^, dictating the molecular formula C_29_H_48_O_5_. The spectroscopic data of metabolite **13** related to the steroidal nucleus (Table 1 and Table 2) displayed high similarity with those of **9**–**12**, suggesting a 3,8,11-trihydroxy-4-methyl steroidal nucleus that was further verified by the correlations observed in the COSY, HMBC and NOESY spectra (Figure 3c). Additionally, the NMR data concerning the side chain of compound **13** were rather similar to those of **8**, thus allowing for the identification of **13** as (23*E*)-22*α*,28-epidioxy-4*α*,24-dimethyl-5*α*-cholest-23-en-3*β*,8*β*,11*β*-triol.

Compound **20**, isolated as a white amorphous solid, had the molecular formula C_28_H_44_O_2_, as indicated by its HR-ESIMS and NMR data. In the ^1^H NMR spectrum of metabolite **20** evident were only one methyl on a non-protonated carbon (*δ*_H_ 0.75), three methyls on tertiary carbons (*δ*_H_ 1.03, 1.04 and 1.06), one hydroxymethylene (*δ*_H_ 3.59 and 3.81), one oxygenated methine (*δ*_H_ 3.56), three olefinic methines (*δ*_H_ 5.56, 5.73 and 5.92) and an exomethylene group (*δ*_H_ 4.79 and 4.83). The spectroscopic data of **20** (Table 1 and Table 2) closely resembled those of the co-occurring **19**, with the main difference being the presence of an additional 1,2-disubstituted double bond in the side chain of **20**. The homonuclear and heteronuclear correlations observed in the COSY, HMBC, and NOESY spectra (Figure 3d) verified the 3,19-dihydroxy steroidal nucleus with a Δ^5^ double bond and the proposed side chain, as well as the relative configuration of the stereogenic centers. The *E* geometry of the Δ^22^ double bond was assigned on the basis of the measured coupling constant between H-22 and H-23 (*J* = 15.7 Hz). On the basis of the above, metabolite **20** was identified as (22*E*)-24-methyl-cholesta-5,22,24(28)-trien-3*β*,19-diol.

Compounds **3**, **5**, **7**, **9**–**12**, **14**–**19,** and **21**–**26** were identified by comparison of their spectroscopic and physical characteristics with those reported in the literature as 4*α*,24-dimethyl-5*α*-cholest-24(28)-en-3*β*-οl (**3**) [6], (22*E*,24*R*)-4*α*,24-dimethyl-5*α*-cholest-22-en-3*β*-ol (**4**) [7], 4*α*,24-dimethyl-5*α*-cholest-24(28)-en-3*β*,8*β*-diol (**5**) [8], 23-oxo-4*α*,24-dimethyl-5*α*-cholest-24(28)-en-3*β*,8*β*-diol (**7**) [9], nebrosteroid M (4*α*,24-dimethyl-5*α*-cholest-24(28)-en-3*β*,8*β*,11*β*-triol, (**9**) [10], (22*E*,24*R*)-4*α*,24-dimethyl-5*α*-cholest-22-en-3*β*,8*β*,11*β*-triol (**10**) [4], nebrosteroid A (23-oxo-4*α*,24-dimethyl-5*α*-cholest-24(28)-en-3*β*,8*β*,11*β*-triol, (**11**) [11], 23*ξ*-acetoxy-4*α*,24-dimethyl-5*α*-cholest-24(28)-en-3*β*,8*β*,11*β*-triol (**12**) [4], (22*Z*)-4*α*,24*ξ*-dimethyl-5*α*-cholest-22-en-3*β*,8*β*,11*β*,12*α*-tetraol (**14**) [4], 4*α*,24-dimethyl-5*α*-cholest-24(28)-en-3*β*,8*β*,18-triol (**15**) [4], (22*E*,24*R*)-4*α*,24-dimethyl-5*α*-cholest-22-en-3*β*,8*β*,18-triol (**16**) [4], 24-methyl-cholesta-5,24(28)-dien-3*β*-οl (**17**) [12], 24-methyl-cholesta-5,24(28)-dien-3*β*,7*β*-diol (**18**) [13], 24-methyl-cholesta-5,24(28)-dien-3*β*,19-diol (**19**) [14], 24-methyl-cholesta-5,24(28)-dien-3*β*,7*β*,19-triol (**21**) [15], 7*β*-acetoxy-24-methyl-cholesta-5,24(28)-dien-3*β*,19-diol (**22**) [16], 7*β*-acetoxy-cholest-5-en-3*β*,19-diol (**23**) [15], 7-oxo-24-methyl-cholesta-5,24(28)-dien-3*β*,19-diol (**24**) [17], 24-methyl-cholesta-7,24(28)-dien-3*β*,5*α*,6*β*-triol (**25**) [18], and (22*E*,24*R*)-24-methyl-cholesta-7,22-dien-3*β*,5*α*,6*β*-triol (**26**) [18], previously isolated from various marine organisms, mainly soft corals of the genera *Litophyton* and *Nephthea*. Even though compound **4** has been isolated in the past, only a few characteristic ^1^H NMR resonances have been reported. Analysis of its 1D and 2D spectra allowed for the full assignment of the ^1^H and ^13^C chemical shifts of compound **4** (Table 1 and Table 2).

### 2.2. Evaluation of the Biological Activity of the Isolated Metabolites

Compounds **3**–**7**, **9**–**12**, **14**–**20,** and **22**–**26**, which were isolated in sufficient amounts, were evaluated in vitro in human tumor and non-cancerous cell lines for a number of biological activities, including cytotoxicity, anti-inflammatory, anti-angiogenic, and neuroprotective activity, as well as for their effect on androgen receptor (AR)-regulated transcription.

Initially, the cytotoxic activity of metabolites **3**–**7**, **9**–**12**, **14**–**20,** and **22**–**26** was evaluated in human cancer and normal cells after 72 h of treatment. Human cervical cancer (HeLa), human breast adenocarcinoma (MCF7) cell lines, and human normal fibroblasts (BJ) were used for the screening. Among the tested compounds, **22** and **23** strongly reduced the viability of cancer cells in the low micromolar range (Table 3), compounds **9**–**12**, **14**–**16**, **18,** and **24** showed moderate cytotoxic activity, while the remaining ten steroids were proven inactive. Most of the compounds with activity against cancer cells also showed cytotoxicity toward normal cells (BJ), except for compounds **11**, **12**, **14**, **15,** and **18**. Compared to cisplatin, these compounds exhibit a wide therapeutic window because of the absence of cytotoxicity on normal human fibroblasts. Moreover, metabolite **22** was proven more active against the HeLa cell line than the reference standard cisplatin.

**Figure 4:**
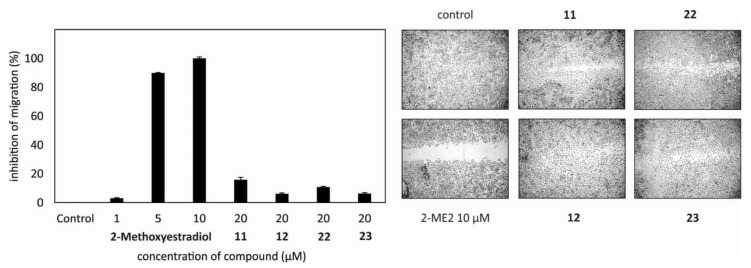
Compounds **11**, **12**, **22,** and **23** inhibited migration of HUVECs after 20 h of treatment at 20 µM. 2-Methoxy-estradiol was used as a positive control. The experiment was repeated three times in triplicates.

Subsequently, we examined whether the isolated steroids could influence angiogenesis or inflammation in vitro. Compounds **3**–**7**, **9**–**12**, **14**–**20,** and **22**–**26** and 2-methoxy-estradiol, which is known as an anti-angiogenic drug for the treatment of tumors and was used herein as positive control [19], were tested in the migration scratch and the tube formation assays using human umbilical vein endothelial cells (HUVEC). Only non-cytotoxic concentrations in HUVECs were used for the scratch assay. Metabolites **11**, **12**, **22,** and **23** partially inhibited HUVEC migration at 20 µM after 20 h of treatment (Figure 4), while in the tube formation assay, where HUVECs may form tube-like structures, no activity was observed for the tested compounds (data not shown). Thus, all tested steroids showed either little or no antiangiogenic activity.

The anti-inflammatory properties of metabolites **3**–**7**, **9**–**12**, **14**–**20,** and **22**–**26** were determined by measuring the levels of endothelial leukocyte adhesion molecule-1 (ELAM/E-selectin), which is a key molecular marker in the initiation of inflammation, expressed on the cell surface. Cell adhesion molecules (ICAM-1, VCAM-1, E-selectin) are significantly increased on the vascular endothelium activated by pro-inflammatory mediators (tumor necrosis factor α, TNFα) as a crucial step for the extravasation of leukocytes into inflamed tissue [20]. TNFα stimulates NFκB (nuclear factor kappa-light-chain-enhancer of activated B cells) and thus E-selectin (CD62E, ELAM). Endothelial cells were pre-treated for 30 min with the tested compounds and then activated with TNFα for 4 h. Curcumin, which inhibits activation of NF-κB and thus inhibits expression and activity of the COX-2 gene induced by TNFα [21], was used as a positive control, decreasing ELAM production to 25% at 10 µM. None of the tested compounds decreased the levels of ELAM (Supplementary Materials Figure S1).

**Figure 5:**
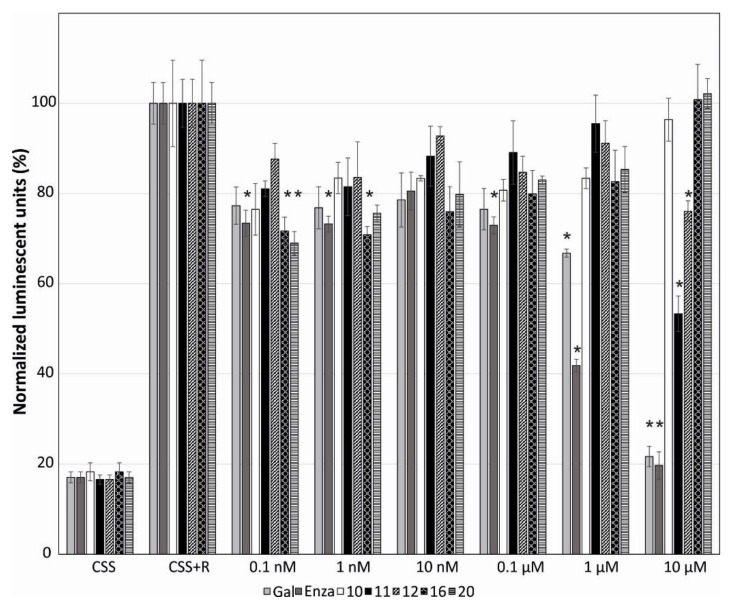
The influence of compounds **10**, **11**, **12**, **16,** and **20** on the androgen receptor-mediated transcription in the 22Rv1-ARE14 reporter cell line. Control cells were grown in charcoal-stripped serum medium (CSS). Cells were stimulated with either 1 nM methyltrienolone R1881 (R) or with the tested compounds in six different concentrations for 24 h in CSS. The luciferase activity was measured in the cell lysate. Enzalutamide (Enza) and galeterone (Gal) were used as positive controls. The experiment was repeated three times in triplicates. Bars with asterisk (*) are significantly different from the control (CSS+R) based on Tukey’s multiple comparison test (*p* ≤ 0.05).

We have previously observed that several steroids with bulky or long side chains, such as galeterone derivatives or cholestanes, can also inhibit AR [22,23]. Therefore, we further analyzed the influence of the isolated steroids on AR-mediated transcription. Compounds **3**–**7**, **9**–**12**, **14**–**20,** and **22**–**26** in six different concentrations were evaluated on the reporter cell line 22Rv1-ARE14 for 24 h. Galeterone and enzalutamide, which were used as positive controls, showed a strong dose-dependent reduction of AR-regulated transcription (22% and 20% inhibition at 10 µM, respectively). Compounds **11**, **12**, **16,** and **20** showed clearly reduced luciferase activity in a dose-dependent manner, already at submicromolar concentrations (Figure 5). The strongest inhibition of AR was displayed by compound **11** at 10 µM. Surprisingly, steroids **10**, **16,** and **20** showed increased inhibition of AR (or decreased the luciferase activity) with decreasing concentrations (Figure 5). All other steroids tested inhibited AR by no more than 20% (data not shown).

**Figure 6:**
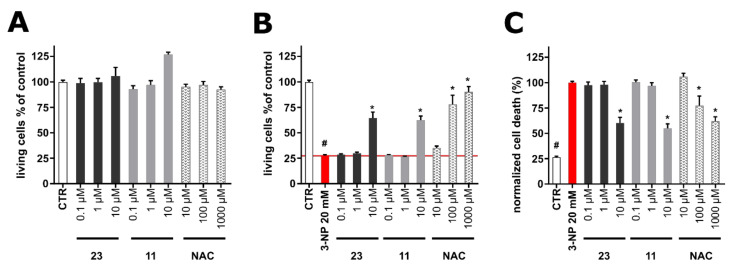
(**A**) Cytotoxicity of compounds **11** and **23** in human neuron-like SH-SY5Y cells after 48 h of treatment. All results are presented as mean ± standard error of the mean (SEM) from at least three independent experiments in triplicates. (**B**) Neuroprotective activity of compounds **11** and **23** in the 3-nitropropionic acid (3-NPA)-induced model of Huntington’s disease on human neuron-like SH-SY5Y cells after 48 h of treatment. (**C**) Cell death of neuron-like SH-SY5Y induced by 3-NPA and the protective effect of compounds **11** and **23** after 48 h. The results are presented as mean ± standard error of the mean (SEM) from triplicates in four independent experiments (*n* = 4). *N*-acetylcysteine (NAC) was used as a positive control. * *p* compared with vehicle with 20 mM 3-NPA, # *p* compared with vehicle without 20 mM 3-NPA.

Since anti-androgens have shown neuroprotective effects in the in vivo model of Huntington’s disease [24], selected compounds that showed cytotoxic activity and moderate activity in the migration scratch assay were tested for their cytotoxicity on differentiated human SH-SY5Y cells (neuron-like cells). Specifically, based on the combined results obtained for compounds **3**–**7**, **9**–**12**, **14**–**20,** and **22**–**26** in the cytotoxicity assay, the anti-inflammatory activity assay, the migration scratch assay, and the tube formation assay in HUVECs, as well as in the reporter assay with AR, compounds **11** and **23** were selected as the two most active compounds that were subsequently evaluated for their neuroprotective activity. As shown in Figure 6A, compounds **11** and **23**, as well as *N*-acetylcystein (NAC) that was used as a positive control, did not show cytotoxic, but rather stimulatory activity. In order to evaluate the potential neuroprotective effect of the compounds, neuroblastoma cell line SH-SY5Y was differentiated for 48 h and further exposed to 20 mM 3-nitropropionic acid (3-NPA) as an agent mimicking Huntington’s disease in vitro [25]. 3-NPA was used alone or in co-treatment with the tested compounds at concentrations of 0.1–10 µM. As shown in Figure 6B, 3-NPA caused dramatic (approx. 70%) decrease in cell viability determined by the Calcein AM assay. The positive control (NAC) showed partial (cell viability 77.9 ± 8.99% at 100 µM) or almost complete (cell viability 90.2 ± 5.28% at 1000 µM) protection of cells from the negative effect of 3-NPA. Compounds **11** and **23** showed significant protective effects at 10 µM (cell viability 64.4 ± 6.02% for **11** and 62.5 ± 3.95% for **23**), comparable to 100 µM of the positive control NAC. Being encouraged by the promising results, we further analyzed the protective effects of **11** and **23** using an orthogonal method (propidium iodide (PI) assay) to verify their activity. In general, PI as a positively charged dye is associated with an increase of cell damage or death, since it penetrates cells with cell-disrupted membranes [26]. Within the 3-NPA model, its toxic effect was considered as 100% of the PI signal and thus reduction of cell death was determined. As shown in Figure 6C, compounds **11** and **23** significantly reduced cell death (maximal effect at 10 µM) in a manner similar to NAC (100 µM, 77.2 ± 9.53%; 1000 µM, 62.1 ± 4.19%), thus confirming the protective effects of **11** and **23** observed in the viability assay.

## 3. Materials and Methods

### 3.1. General Experimental Procedures

Optical rotations were measured on a Krüss polarimeter (A. KRÜSS Optronic GmbH, Hamburg, Germany) equipped with a 0.5 dm cell. UV spectra were recorded on a Lambda 40 UV/Vis spectrophotometer (Perkin Elmer Ltd., Beaconsfield, UK). IR spectra were obtained on an Alpha II FTIR spectrometer (Bruker Optik GmbH, Ettlingen, Germany). Low-resolution EI mass spectra were measured on a Thermo Electron Corporation DSQ mass spectrometer (Thermo Fisher Scientific, Bremen, Germany). High-resolution APCI or ESI mass spectra were measured on a LTQ Orbitrap Velos mass spectrometer (Thermo Fisher Scientific, Bremen, Germany). NMR spectra were recorded on Bruker AC 200, DRX 400, and Avance NEO 950 (Bruker BioSpin GmbH, Rheinstetten, Germany) and Varian 600 (Varian, Inc., Palo Alto, CA, USA) spectrometers. Chemical shifts are given on the *δ* (ppm) scale with reference to the solvent signals. The 2D NMR experiments (HSQC, HMBC, COSY, NOESY) were performed using standard Bruker or Varian pulse sequences. Column chromatography separations were performed with Kieselgel 60 (Merck, Darmstadt, Germany). HPLC separations were conducted on a Waters 600 liquid chromatography pump equipped with a Waters 410 differential refractometer (Waters Corporation, Milford, MA, USA), using a Kromasil 100 C_18_ (25 cm × 8 mm i.d.) column (MZ-Analysentechnik GmbH, Mainz, Germany). TLC were performed with Kieselgel 60 F_254_ aluminum plates (Merck, Darmstadt, Germany) and spots were detected after spraying with 25% H_2_SO_4_ in MeOH reagent and heating at 100 °C for 1 min.

### 3.2. Biological Material

Specimens of *S. polydactyla* were hand-picked by SCUBA diving at a depth of 10 m from the reefs near the National Institute of Oceanography and Fisheries (NOIF), Hurghada, Egypt (GPS coordinates 27°17’06’’N, 33°46’24’’E) in June 2015 and transported to the laboratory in ice chests, where they were stored at −20 °C until analyzed. A voucher specimen has been deposited at the animal collection of NOIF in Hurghada and the animal collection of the Section of Pharmacognosy and Chemistry of Natural Products, Department of Pharmacy, National and Kapodistrian University of Athens (ATPH/MP0533).

### 3.3. Extraction and Isolation

Specimens of the freeze-dried gorgonian (119.9 g) were exhaustively extracted with mixtures of CH_2_Cl_2_/MeOH (2:1) at room temperature. Evaporation of the solvents under vacuum afforded a dark green residue (18.5 g) which was submitted to vacuum column chromatography on silica gel using cHex with increasing amounts of EtOAc followed by EtOAc with increasing amounts of MeOH as eluent to yield 9 fractions (A–I). Fraction B (2.5 g, 30–50% EtOAc in cHex) was fractionated by vacuum column chromatography on silica gel using mixtures of cHex/EtOAc of increasing polarity as mobile phase to yield 6 fractions (B1–B6). Fraction B6 (0.5 g, 20–30% EtOAc in cHex) was further fractionated by gravity column chromatography on silica gel using mixtures of cHex/EtOAc of increasing polarity as mobile phase to afford 10 fractions (B6a–B6j). Fractions B6e, B6g, and B6h were subjected repeatedly to reversed-phase HPLC, using MeOH/H_2_O (100:0 and 98:2) as eluent to yield compounds **3** (9.9 mg), **4** (8.8 mg), **5** (1.8 mg), **6** (5.9 mg), **8** (0.8 mg), and **17** (14.0 mg). Fraction C (2.5 g, 60–70% EtOAc in cHex) was submitted to vacuum column chromatography on silica gel using mixtures of cHex/EtOAc of increasing polarity as mobile phase to afford 9 fractions (C1–C9). Fractions C5, C6, C7, and C8 were subjected repeatedly to reversed-phase HPLC using MeOH/H_2_O (100:0, 98:2, and 97:3) as eluent to afford compounds **1** (0.7 mg), **2** (0.3 mg), **7** (6.3 mg), **9** (5.7 mg), and **10** (5.7 mg). Fraction D (0.9 g, 80–90% EtOAc in cHex) was fractionated by gravity column chromatography on silica gel using cHex with increasing amounts of Me_2_CO as mobile phase to afford 18 fractions (D1–D18). Fractions D8, D9, D11, and D12 were subjected repeatedly to reversed-phase HPLC using MeOH/H_2_O (100:0 and 98:2) as eluent to yield compounds **11** (4.0 mg), **12** (2.2 mg), **13** (1.3 mg), **14** (1.1 mg), **15** (9.4 mg), and **16** (2.3 mg). Fraction E (0.23 g, 100% EtOAc) was fractionated by gravity column chromatography on silica gel using cHex with increasing amounts of Me_2_CO as mobile phase to afford 10 fractions (E1–E10). Fractions E3, E5, and E6 were subjected repeatedly to reversed-phase HPLC using mixtures of MeOH/H_2_O (100:0 and 97:3) as eluent to yield compounds **18** (1.7 mg), **19** (17.5 mg), **20** (2.0 mg), **22** (2.1 mg), and **23** (1.0 mg). Fraction G (1.7 g, 50% MeOH in EtOAc) was separated by vacuum column chromatography on silica gel using cHex with increasing amounts of EtOAc and EtOAc with increasing amounts of MeOH to yield 7 fractions (G1–G7). Fractions G5 and G7 were subjected repeatedly to reversed-phase HPLC using MeOH/H_2_O (100:0 and 97:3) as eluent to yield compounds **24** (6.9 mg), **25** (2.0 mg), and **26** (3.1 mg). Fraction I (5.1 g, 100% MeOH) was subjected to vacuum column chromatography on silica gel using cHex with increasing amounts of EtOAc followed by EtOAc with increasing amounts of MeOH to afford 11 fractions (I1–I11). Fractions I6 and I7 were combined and subjected repeatedly to reversed-phase HPLC using MeOH (100%) as eluent to yield compound **21** (2.3 mg).

(9*E*,22*E*,24*R*)-3*β*-Hydroxy-4*α*,24-dimethyl-8,9-seco-5α-cholesta-9(11),22-dien-8-one (**1**): White amorphous solid; ^1^H and ^13^C NMR data, see Table 1 and Table 2; HR-APCIMS *m/z* 429.3724 [M + H]^+^ (calcd. for C_29_H_49_O_2_, 429.3727).

(9*E*)-3*β*-Hydroxy-4*α*,24-dimethyl-8,9-seco-5α-cholesta-9(11),24(28)-dien-8-one (**2**): White amorphous solid; ^1^H NMR data, see Table 1; HR-APCIMS *m*/*z* 429.3725 [M + H]^+^ (calcd. for C_29_H_49_O_2_, 429.3727).

(22*E*,24*R*)-4*α*,24-Dimethyl-5*α*-cholest-22-en-3*β*,8*β*-diol (**6**): White amorphous solid; [α${]}_{D}^{20}$ + 68 (*c* 0.25, CHCl_3_); UV (CHCl_3_) *λ*_max_ (log *ε*) 206 (2.85); IR (thin film) *ν*_max_ 3440, 2954, 2856, 1466, 1384, 964 cm^−1^; ^1^H and ^13^C NMR data, see Table 1 and Table 2; HR-APCIMS *m*/*z* 413.3773 [M − H_2_O + H]^+^ (calcd. for C_29_H_49_O, 413.3778).

(23*E*)-22*α*,28-Epidioxy-4*α*,24-dimethyl-5*α*-cholest-23-en-3*β*,8*β*-diol (**8**): White amorphous solid; [α${]}_{D}^{20}$ −50 (*c* 0.02, CHCl_3_); UV (CHCl_3_) *λ*_max_ (log *ε*) 206 (3.94), 225 (3.84); IR (thin film) *ν*_max_ 3458, 2927, 2861, 1723, 1462, 1378, 1243, 1013 cm^−1^; ^1^H and ^13^C NMR data, see Table 1 and Table 2; HR-APCIMS *m*/*z* 459.3475 [M − H]^−^ (calcd. for C_29_H_47_O_4_, 459.3480).

(23*E*)-22*α*,28-Epidioxy-4*α*,24-dimethyl-5*α*-cholest-23-en-3*β*,8*β*,11*β*-triol (**13**): White amorphous solid; [α${]}_{D}^{20}$ +30 (*c* 0.1, CHCl_3_); UV (CHCl_3_) *λ*_max_ (log *ε*) 206 (3.26), 230 (2.97); IR (thin film) *ν*_max_ 3403, 2923, 2873, 1725, 1450, 1377, 1261, 1045 cm^−1^; ^1^H and ^13^C NMR data, see Table 1 and Table 2; HR-APCIMS *m/z* 475.3424 [M − H]^−^ (calcd. for C_29_H_47_O_5_, 475.3429).

(22*E*)-24-Methyl-cholesta-5,22,24(28)-trien-3*β*,19-diol (**20**): White amorphous solid; [α${]}_{D}^{20}$ −133 (*c* 0.03, CHCl_3_); UV (CHCl_3_) *λ*_max_ (log *ε*) 206 (3.61), 232 (3.76); IR (thin film) *ν*_max_ 3433, 2919, 2857, 1462, 1377, 1261, 1037 cm^−1^; ^1^H and ^13^C NMR data, see Table 1 and Table 2; HR-ESIMS *m*/*z* 411.3271 [M − H]^−^ (calcd. for C_28_H_43_O_2_, 411.3269).

### 3.4. Cell Culture

The tested compounds were dissolved in DMSO to afford 10 mM stock solutions. Human cervical carcinoma (HeLa) and human breast adenocarcinoma (MCF7) cell lines were purchased from European Collection of Authenticated Cell Cultures (ECACC, Salisbury, UK) and cultivated in Dulbecco’s Modified Eagle Medium (DMEM) (Merck, Darmstadt, Germany), as previously reported [27]. Human umbilical vein endothelial cells (HUVECs) were a kind gift of Prof. Jitka Ulrichová (Faculty of Medicine and Dentistry, Palacky University, Olomouc, Czech Republic). The cultivation and assay was performed in endothelial cell proliferation medium (ECPM, Provitro, Berlin, Germany) [23]. The 22Rv1-ARE14 reporter cell line [28] was a generous gift of Prof. Zdeněk Dvořák (Department of Cell Biology and Genetics, Palacky University). The 22Rv1-ARE14 cell line was grown in Roswell Park Memorial Institute 1640 Medium (RPMI 1640) [22]. All cells were maintained in a humidified CO_2_ incubator at 37 °C using the standard trypsinization procedure twice or three times a week. The SH-SY5Y human neuroblastoma cell line (ECACC, Salisbury, UK) was cultivated in DMEM and Ham’s F12 Nutrient Mixture (DMEM:F12, 1:1), as previously described [25]. Cells were used up to twenty passages. All trans-retinoic acid (10 µM) in 1% FBS DMEM/F12 medium was added to SH-SY5Y cells to achieve differentiation conditions [29,30], to reach longer neurites and reduced proliferation (48 h). All cells were maintained in a humidified CO_2_ incubator at 37 °C using the standard trypsinization procedure twice or three times a week.

### 3.5. Evaluation of Cytotoxicity

In cytotoxicity assays, cancer cells were treated with six different concentrations of each tested compound for 72 h. Cells were stained with resazurin and IC_50_ values were calculated as previously reported [26]. Triplicates from at least three independent experiments were used. For the ELAM assay, the Calcein AM (Molecular Probes, Invitrogen, Karlsruhe, Germany) cytotoxicity assay, which assessed HUVEC viability after 4 h treatment, was used as previously described [31].

### 3.6. Cell-Surface ELISA CD62E (E-Selectin, ELAM)

An enzyme-linked immunosorbent assay (ELISA) was used to detect the levels of the cell adhesion molecule ELAM in HUVEC cells after 30 min of incubation with the tested compounds and 4 h of stimulation with TNFα, as previously described [31].

### 3.7. Migration Scratch Assay

The scratch test was performed with HUVEC cells and evaluated after 20 h of treatment, as previously reported [23].

### 3.8. AR-Transcriptional Reporter Assay

AR-transcriptional reporter assays were performed on 22Rv1-ARE14 cells after 24 h of incubation, as previously described [22].

### 3.9. SH-SY5Y Cell Treatments and Evaluation of Cell Viability/Cytotoxicity

Cell viability of neuron-like SH-SY5Y cells growing in 96-well plates (7000 cells/well) 24 h after treatment was evaluated using the Calcein AM assay [27], with minor modification of Calcein AM concentration (0.75 µM). Cell death of SH-SY5Y cells (20,000 cells/well) was determined using the propidium iodide (PI) assay according to Stone et al. with a slight modification [32]. Briefly, PI solution in PBS is added to cell medium to reach concentration 1 µg/mL, incubated for 15 min at room temperature and quantified at 535/617 nm (excitation/emission) by Infinite M200 Pro reader (Tecan, Austria). The resulting fluorescence of 3-NPA toxin was considered as 100% cell death. In the assays, neuron-like cells were treated with the tested compounds in 0.1–10 µM concentration range for 48 h. DMSO-treated cells (≤0.1% *v*/*v*) were used as healthy controls.

### 3.10. Statistical Analysis

All data are expressed as mean ± SD or SEM. Data were evaluated by non-parametric Kruskal–Wallis test followed by post-hoc Mann–Whitney test (Figure 6) with sequential Bonferroni correction of *p*-values or ANOVA followed by Tukey’s multiple comparison test (Figure 5) using the PAST (version 1.97) software package [33]. *p*-values < 0.05 were considered statistically significant.

## 4. Conclusions

The chemical analysis of the organic extract of the soft coral *S. polydactyla* collected from the Hurghada reef in the Red Sea resulted in the isolation of 26 steroids, with six of them (**1**, **2**, **6**, **8**, **13,** and **20**) being new natural products. Among them, **1** and **2** display the rare 8,9-seco-cholestane steroidal nucleus. To the best of our knowledge, dictyoneolone, isolated from a sponge of the genus *Dictyonella*, is the only previously reported 4-methyl-8,9-seco-cholestane derivative [34]. Evaluation of cytotoxic, anti-inflammatory, anti-angiogenic, and neuroprotective activity of the majority of the isolated metabolites that were isolated in adequate quantities revealed significant cytotoxicity in the low micromolar range against the HeLa and MCF7 cancer cell lines for compounds **22** and **23**, while compounds **11**, **12**, **22,** and **23** inhibited the migration of endothelial cells at 20 µM. Most of the compounds with activity against cancer cells also showed cytotoxicity toward normal cells (BJ), except for compounds **11**, **12**, **14**, **15,** and **18**. Compared to cisplatin, these compounds have therefore a broad therapeutic window due to low or zero cytotoxicity on normal human fibroblasts. Moreover, metabolite **22** was more active against HeLa cells than cisplatin used as positive control. Furthermore, the effect of the isolated metabolites on AR-regulated transcription was evaluated in vitro in human tumor and non-cancerous cells, with compound **11** exhibiting the strongest inhibition of AR at 10 µM. It is worth-noting that metabolites **10**, **16,** and **20** displayed increased inhibition of AR with decreasing concentrations. In addition, compounds **11** and **23** showed neuroprotective activity on neuron-like SH-SY5Y cells.

## Figures and Tables

**Table 1:** ^1^H NMR data (*δ* in ppm, *J* in Hz) in CDCl_3_ of compounds **1**, **2**, **4**, **6**, **8**, **13,** and **20**.

Position	1 ^1^	2 ^1^	4 ^2^	6 ^2^	8 ^1^	13 ^1^	20 ^3^
1	1.33 m, 1.22 m	1.36 m, 1.24 m	1.71 m, 0.98 m	1.72 m, 0.93 m	1.72 m, 0.96 m	1.91 m, 1.00 m	1.91 m, 1.08 m
2	1.76 m, 1.48 m	1.76 m, 1.49 m	1.77 m, 1.46 m	1.77 m, 1.52 m	1.78 m, 1.52 m	1.79 m, 1.58 m	1.84 m, 1.40 m
3	3.09 td (10.1, 4.8)	3.10 m	3.06 td (10.5, 4.9)	3.04 td (10.2, 4.9)	3.06 td (10.5, 5.2)	3.05 td (10.8, 5.0)	3.56 m
4	1.27 m	1.28 m	1.27 m	1.31 m	1.33 m	1.41 m	2.37 m, 2.18 m
5	0.91 m	0.92 m	0.72 m	0.68 td (12.4, 2.9)	0.71 td (12.2, 2.2)	0.69 td (11.9, 2.2)	-
6	1.66 m, 1.60 m	1.66 m, 1.62 m	1.64 m, 1.47 m	1.50 m, 1.33 m	1.53 m, 1.33 m	1.56 m, 1.39 m	5.73 m
7	2.29 m, 1.75 m	2.48 m, 2.24 m	1.69 m, 1.50 m	1.63 m, 1.13 m	1.64 m, 1.19 m	1.71 m, 1.22 m	2.01 m, 1.51 m
8	-	-	1.28 m	-	-	-	1.82 m
9	5.28 d (15.3)	5.27 m	0.60 m	0.80 m	0.81 m	0.88 m	0.89 m
11	5.41 ddd (15.3, 11.1, 3.8)	5.41 m	1.47 m, 1.00 m	1.62 m, 1.49 m	1.62 m, 1.48 m	4.43 brd (1.9)	1.62 m, 1.53 m
12	2.47 m, 1.70 m	2.50 m, 1.72 m	1.92 m, 1.11 m	1.95 m, 1.16 m	1.93 m, 1.17 m	2.23 m, 1.37 m	2.01 m, 1.17 m
14	2.49 m	2.50 m	0.93 m	1.18 m	1.23 m	1.27 m	0.90 m
15	1.64 m, 1.48 m	1.64 m, 1.50 m	1.51 m, 1.02 m	1.45 m, 1.23 m	1.53 m, 1.33 m	1.60 m, 1.46 m	1.50 m, 1.05 m
16	1.70 m, 1.47 m	1.73 m, 1.49 m	1.63 m, 1.19 m	1.64 m, 1.23 m	1.99 m, 1.31 m	2.00 m, 1.35 m	1.64 m, 1.26 m
17	1.31 m	1.28 m	1.09 m	1.03 m	1.43 m	1.43 m	1.18 m
18	1.08 s	1.09 s	0.64 s	0.92 s	0.89 s	1.09 s	0.75 s
19	0.94 s	0.94 s	0.80 s	0.97 s	0.96 s	1.33 s	3.81 d (11.5), 3.59 d (11.5)
20	2.10 m	2.11 m	1.98 m	1.96 m	1.56 m	1.59 m	2.11 m
21	0.99 d (6.8)	0.93 d (6.1)	0.97 d (6.6)	0.94 d (6.4)	0.83 d (6.9)	0.85 d (7.0)	1.03 d (6.5)
22	5.15 dd (15.2, 8.1)	1.53 m, 1.14 m	5.12 dd (15.1, 7.6)	5.09 dd (15.2, 8.2)	4.72 brs	4.70 brs	5.56 dd (15.7, 8.7)
23	5.20 dd (15.2, 7.3)	2.08 m, 1.88 m	5.17 dd (15.1, 7.0)	5.16 dd (15.2, 7.4)	5.36 brs	5.36 brs	5.92 d (15.7)
24	1.83 m	-	1.83 m	1.80 m	-	-	-
25	1.44 m	2.21 m	1.44 m	1.42 m	2.24 septet (6.9)	2.24 septet (6.9)	2.53 septet (6.8)
26	0.81 d (6.8)	1.00 d (6.8)	0.81 d (6.8)	0.81 d (6.8)	1.05 d (6.9)	1.05 d (6.9)	1.06 d (6.8)
27	0.80 d (6.8)	1.01 d (6.9)	0.79 d (6.8)	0.79 d (6.8)	1.05 d (6.9)	1.06 d (6.9)	1.04 d (6.8)
28	0.89 d (6.8)	4.70 brs, 4.63 brs	0.88 d (6.8)	0.88 d (6.8)	4.62 d (15.7), 4.20 d (15.7)	4.59 d (15.7), 4.21 d (15.7)	4.83 brs, 4.79 brs
29	1.04 d (6.1)	1.04 d (6.1)	0.92 d (6.3)	0.95 d (6.8)	0.95 d (6.4)	0.96 d (6.4)	-

^1^ Recorded at 600 MHz. ^2^ Recorded at 400 MHz. ^3^ Recorded at 950 MHz, - absence of value.

**Table 2:** ^13^C NMR data (*δ* in ppm) in CDCl_3_ of compounds **1**, **4**, **6**, **8**, **13,** and **20**.

Position	1 ^1,2^	4 ^3^	6 ^1,4^	8 ^1,2^	13 ^2^	20 ^1,5^
1	38.5	36.8	37.7	37.2	37.5	32.9
2	30.7	31.1	30.9	30.3	30.2	31.7
3	76.6	76.6	76.8	76.4	76.5	70.8
4	39.8	39.2	39.0	38.5	38.2	41.9
5	53.5	51.0	51.9	51.2	52.3	134.9
6	20.0	24.2	20.5	19.8	20.0	126.8
7	47.1	32.2	40.1	39.6	39.9	30.8
8	213.1	36.0	73.6	73.5	75.3	33.4
9	142.0	54.6	56.6	56.0	57.6	49.9
10	39.1	34.9	36.9	36.3	36.8	41.0
11	127.6	21.1	18.3	18.1	69.8	21.4
12	46.8	40.2	41.6	40.5	49.0	39.6
13	55.2	42.4	43.0	42.7	41.8	42.3
14	62.7	56.6	59.7	59.0	60.2	57.5
15	26.6	24.2	19.0	18.7	19.2	24.1
16	28.4	28.6	28.3	27.0	26.9	28.1
17	56.7	56.1	57.1	52.4	53.9	55.4
18	13.5	12.3	14.0	12.8	15.0	12.0
19	15.8	13.4	13.8	13.2	15.6	62.3
20	39.1	40.0	39.6	39.8	40.0	39.9
21	21.7	20.9	21.3	12.8	12.9	20.2
22	135.2	135.9	135.7	79.9	79.7	135.4
23	132.6	131.6	131.8	119.4	119.2	128.8
24	43.0	42.8	43.2	142.0	142.0	153.0
25	33.2	33.1	33.8	30.8	31.1	28.8
26	19.8	19.6	19.9	21.0	21.1	21.7
27	20.1	19.9	20.2	21.0	21.1	22.1
28	17.6	17.6	18.0	70.6	70.8	109.0
29	16.4	15.1	16.0	14.9	15.2	

^1^ Chemical shifts were determined through HMBC correlations. ^2^ Recorded at 150 MHz. ^3^ Recorded at 50 MHz. ^4^ Recorded at 100 MHz. ^5^ Recorded at 237.5 MHz.

**Table 3:** Cytotoxicity (IC_50_; µM) of compounds **3**–**7**, **9**–**12**, **14**–**20,** and **22**–**26** against human cancer cell lines and fibroblasts after 72 h of treatment. Cisplatin was used as a positive control.

Compound	HeLa	MCF7	BJ
**3**	>50	>50	>50
**4**	>50	>50	>50
**5**	>50	>50	>50
**6**	>50	>50	>50
**7**	>50	>50	>50
**9**	25.9 ± 4.9	36.4 ± 5.7	18.3 ± 4.0
**10**	32.9 ± 6.8	32.7 ± 1.3	4.1 ± 1.9
**11**	18.8 ± 6.4	21.7 ± 1.4	>50
**12**	15.7 ± 2.0	25.3 ± 6.5	>50
**14**	>50	29.1 ± 5.0	>50
**15**	19.0 ± 4.3	18.9 ± 0.1	>50
**16**	32.9 ± 5.2	33.8 ± 1.0	23.2 ± 1.4
**17**	>50	>50	>50
**18**	22.6 ± 0.4	28.6 ± 5.3	>50
**19**	>50	>50	>50
**20**	>50	>50	>50
**22**	7.5 ± 0.1	8.9 ± 0.0	14.8 ± 5.8
**23**	12.0 ± 1.7	11.2 ± 0.5	14.5 ± 3.9
**24**	21.4 ± 2.0	31.7 ± 0.3	45.9 ± 2.8
**25**	>50	>50	>50
**26**	>50	>50	>50
cisplatin	11.4 ± 3.8	7.7 ± 1.7	6.9 ± 0.9

## Supplementary Materials

The following are available online at https://www.mdpi.com/1660-3397/18/12/632/s1. Figure S1: Visual representation of the evaluation of the activity of the tested metabolites against ELAM. Figures S2–S29: 1D and 2D NMR and HR-MS spectra of compounds **1**, **2**, **6**, **8**, **13,** and **20**.
