# Supplementary material for: Bioactive Steroids from the Red Sea Soft Coral Sinularia polydactyla
Source: Mar Drugs. 2020 Dec 10;18(12):632. doi: 10.3390/md18120632 (PMC7763444; doi:10.3390/md18120632)
Supplement: Supplementary file 1 [file marinedrugs-18-00632-s001.pdf]

Supplementary Materials for

Bioactive Steroids from the Red Sea Soft Coral *Sinularia polydactyla*

Mohamed A. Tammam [1,2], Lucie Rárová [3,*], Marie Kvasnicová [3], Gabriel Gonzalez [3], Ahmed M. Emam [2], Aldoushy Mahdy [4], Miroslav Strnad [3], Efstathia Ioannou [1] and Vassilios Roussis [1,*]

[1] Section of Pharmacognosy and Chemistry of Natural Products, Department of Pharmacy, National and Kapodistrian University of Athens, Panepistimiopolis Zografou, 15771 Athens, Greece; mtammam@pharm.uoa.gr (M.A.T.); eioannou@pharm.uoa.gr (E.I.)

[2] Department of Biochemistry, Faculty of Agriculture, Fayoum University, 63514 Fayoum, Egypt; ame01@fayoum.edu.eg (A.M.E.)

[3] Laboratory of Growth Regulators, Institute of Experimental Botany of the Czech Academy of Sciences, and Faculty of Science, Palacký University, Šlechtitelů 27, Olomouc CZ-78371, Czech Republic; kvasnicova@ueb.cas.cz (M.K.); gonzalez.gabriel@seznam.cz (G.G.); miroslav.strnad@upol.cz (M.S.)

[4] Department of Zoology, Faculty of Science, Al-Azhar University (Assiut Branch), 71524 Assiut, Egypt; aldoushy@azhar.edu.eg (A.M.)

* Correspondence: lucie.rarova@upol.cz (L.R.); roussis@pharm.uoa.gr (V.R.); Tel.: +420-5856-34850698 (L.R.); +30-210-727-4592 (V.R.)

Table of Contents

Figure S1. Compounds **3**–**7**, **9**–**12**, **14**–**20** and **22**–**26** did not affect the relative amount of E-selectin on the cell surface of endothelial cells that were pretreated with 10 μM compounds for 30 min and subsequently stimulated with 10 ng/ml TNF*α* for 4 h. Data are means ± SDs (*n* = 3). S3
Figure S2. ^{1}H NMR spectrum of (9*E*,22*E*,24*R*)-3*β*-hydroxy-4*α*,24-dimethyl-8,9-seco-5*α*-cholesta-9(11),22-dien-8-one (**1**) in $CDCl_3$. S4
Figure S3. HSQC spectrum of (9*E*,22*E*,24*R*)-3*β*-hydroxy-4*α*,24-dimethyl-8,9-seco-5*α*-cholesta-9(11),22-dien-8-one (**1**) in $CDCl_3$. S4
Figure S4. HMBC spectrum of (9*E*,22*E*,24*R*)-3*β*-hydroxy-4*α*,24-dimethyl-8,9-seco-5*α*-cholesta-9(11),22-dien-8-one (**1**) in $CDCl_3$. S5
Figure S5. COSY spectrum of (9*E*,22*E*,24*R*)-3*β*-hydroxy-4*α*,24-dimethyl-8,9-seco-5*α*-cholesta-9(11),22-dien-8-one (**1**) in $CDCl_3$. S5
Figure S6. HR-APCIMS spectrum of (9*E*,22*E*,24*R*)-3*β*-hydroxy-4*α*,24-dimethyl-8,9-seco-5*α*-cholesta-9(11),22-dien-8-one (**1**). S6
Figure S7. ^{1}H NMR spectrum of (9*E*)-3*β*-hydroxy-4*α*,24-dimethyl-8,9-seco-5*α*-cholesta-9(11),24(28)-dien-8-one (**2**) in $CDCl_3$. S6
Figure S8. COSY spectrum of (9*E*)-3*β*-hydroxy-4*α*,24-dimethyl-8,9-seco-5*α*-cholesta-9(11),24(28)-dien-8-one (**2**) in $CDCl_3$. S7
Figure S9. HR-APCIMS spectrum of (9*E*)-3*β*-hydroxy-4*α*,24-dimethyl-8,9-seco-5*α*-cholesta-9(11),24(28)-dien-8-one (**2**). S7
Figure S10. ^{1}H NMR spectrum of (22*E*,24*R*)-4*α*,24-dimethyl-5*α*-cholest-22-en-3*β*,8*β*-diol (**6**) in $CDCl_3$. S8
Figure S11. HSQC spectrum of (22*E*,24*R*)-4*α*,24-dimethyl-5*α*-cholest-22-en-3*β*,8*β*-diol (**6**) in $CDCl_3$. S8
Figure S12. HMBC spectrum of (22*E*,24*R*)-4*α*,24-dimethyl-5*α*-cholest-22-en-3*β*,8*β*-diol (**6**) in $CDCl_3$. S9
Figure S13. COSY spectrum of (22*E*,24*R*)-4*α*,24-dimethyl-5*α*-cholest-22-en-3*β*,8*β*-diol (**6**) in $CDCl_3$. S9
Figure S14. HR-APCIMS spectrum of (22*E*,24*R*)-4*α*,24-dimethyl-5*α*-cholest-22-en-3*β*,8*β*-diol (**6**). S10
Figure S15. ^{1}H NMR spectrum of (23*E*)-22*α*,28-epidioxy-4*α*,24-dimethyl-5*α*-cholest-23-en-3*β*,8*β*-diol (**8**) in $CDCl_3$. S10
Figure S16. HSQC spectrum of (23*E*)-22*α*,28-epidioxy-4*α*,24-dimethyl-5*α*-cholest-23-en-3*β*,8*β*-diol (**8**) in $CDCl_3$. S11
Figure S17. HMBC spectrum of (23*E*)-22*α*,28-epidioxy-4*α*,24-dimethyl-5*α*-cholest-23-en-3*β*,8*β*-diol (**8**) in $CDCl_3$. S11
Figure S18. COSY spectrum of (23*E*)-22*α*,28-epidioxy-4*α*,24-dimethyl-5*α*-cholest-23-en-3*β*,8*β*-diol (**8**) in $CDCl_3$. S12
Figure S19. HR-APCIMS spectrum of (23*E*)-22*α*,28-epidioxy-4*α*,24-dimethyl-5*α*-cholest-23-en-3*β*,8*β*-diol (**8**). S12
Figure S20. ^{1}H NMR spectrum of (23*E*)-22*α*,28-epidioxy-4*α*,24-dimethyl-5*α*-cholest-23-en-3*β*,8*β*,11*β*-triol (**13**) in $CDCl_3$. S13
Figure S21. HSQC spectrum of (23*E*)-22*α*,28-epidioxy-4*α*,24-dimethyl-5*α*-cholest-23-en-3*β*,8*β*,11*β*-triol (**13**) in $CDCl_3$. S13
Figure S22. HMBC spectrum of (23*E*)-22*α*,28-epidioxy-4*α*,24-dimethyl-5*α*-cholest-23-en-3*β*,8*β*,11*β*-triol (**13**) in $CDCl_3$. S14
Figure S23. COSY spectrum of (23*E*)-22*α*,28-epidioxy-4*α*,24-dimethyl-5*α*-cholest-23-en-3*β*,8*β*,11*β*-triol (**13**) in $CDCl_3$. S14
Figure S24. HR-APCIMS spectrum of (23*E*)-22*α*,28-epidioxy-4*α*,24-dimethyl-5*α*-cholest-23-en-3*β*,8*β*,11*β*-triol (**13**). S15
Figure S25. ^{1}H NMR spectrum of (22*E*)-24-methyl-cholesta-5,22,24(28)-trien-3*β*,19-diol (**20**) in $CDCl_3$. S15
Figure S26. HSQC spectrum of (22*E*)-24-methyl-cholesta-5,22,24(28)-trien-3*β*,19-diol (**20**) in $CDCl_3$. S16
Figure S27. HMBC spectrum of (22*E*)-24-methyl-cholesta-5,22,24(28)-trien-3*β*,19-diol (**20**) in $CDCl_3$. S16
Figure S28. COSY spectrum of (22*E*)-24-methyl-cholesta-5,22,24(28)-trien-3*β*,19-diol (**20**) in $CDCl_3$. S17
Figure S29. HR-ESIMS spectrum of (22*E*)-24-methyl-cholesta-5,22,24(28)-trien-3*β*,19-diol (**20**). S17

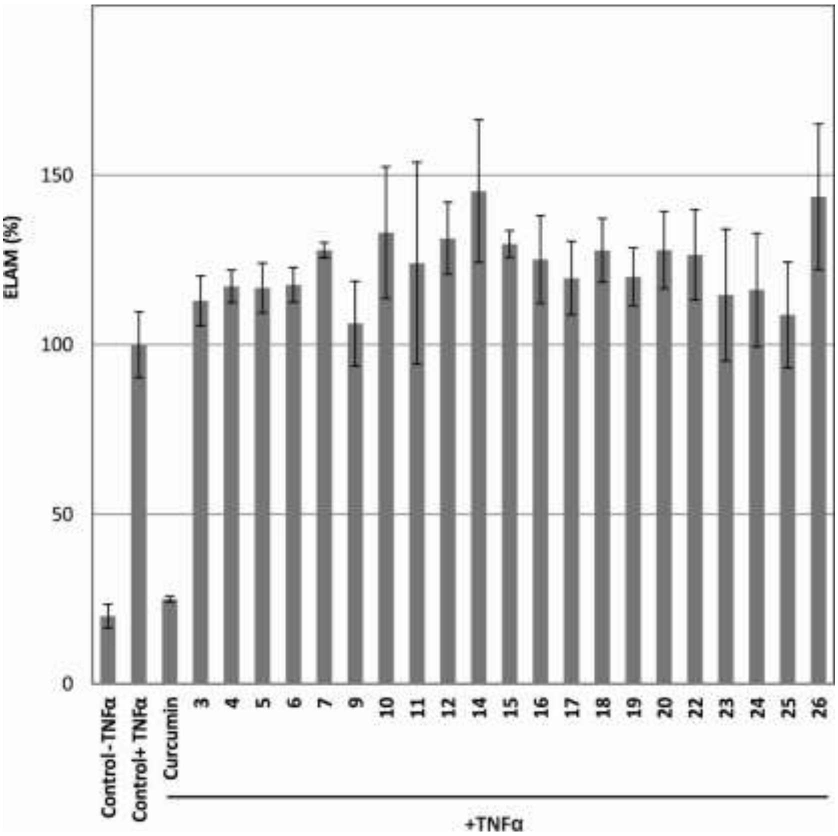

Figure S1. Compounds **3–7**, **9–12**, **14–20** and **22–26** did not affect the relative amount of E-selectin on the cell surface of endothelial cells that were pretreated with 10 μM compounds for 30 min and subsequently stimulated with 10 ng/ml TNF*α* for 4 h. Data are means ± SDs (*n* = 3).

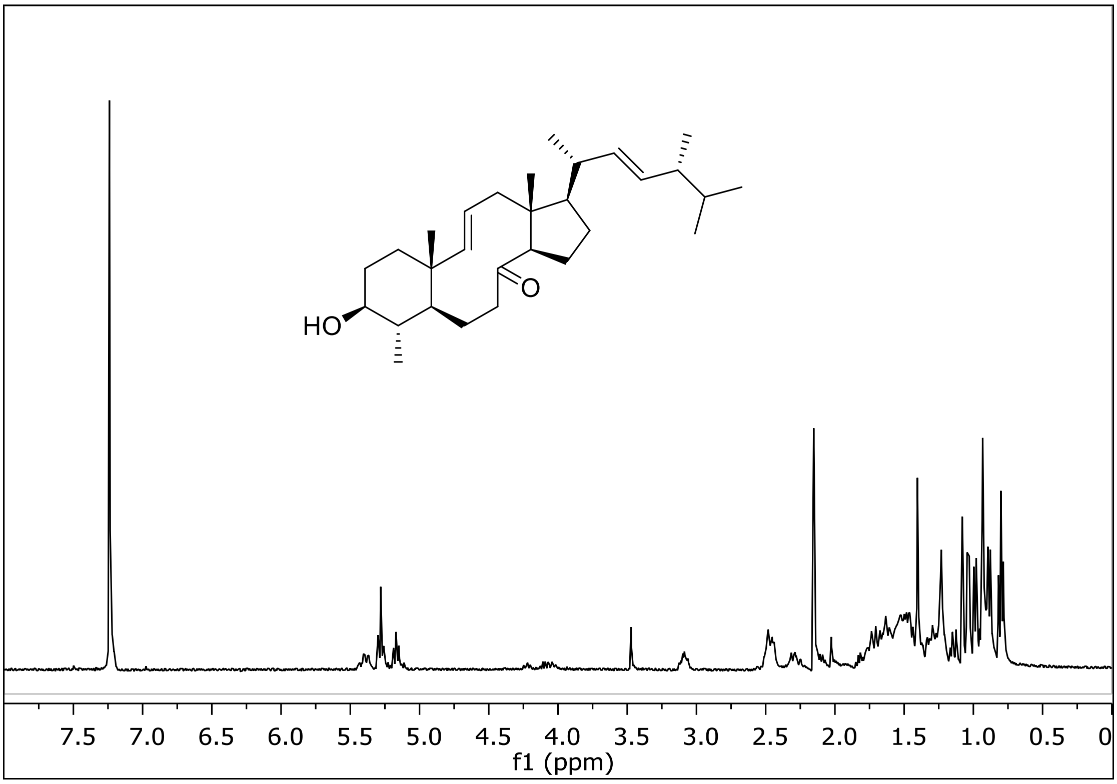

Figure S2. ^{1}H NMR spectrum of (9*E*,22*E*,24*R*)-3*β*-hydroxy-4*α*,24-dimethyl-8,9-seco-5*α*-cholesta-9(11),22-dien-8-one (**1**) in $CDCl_3$.

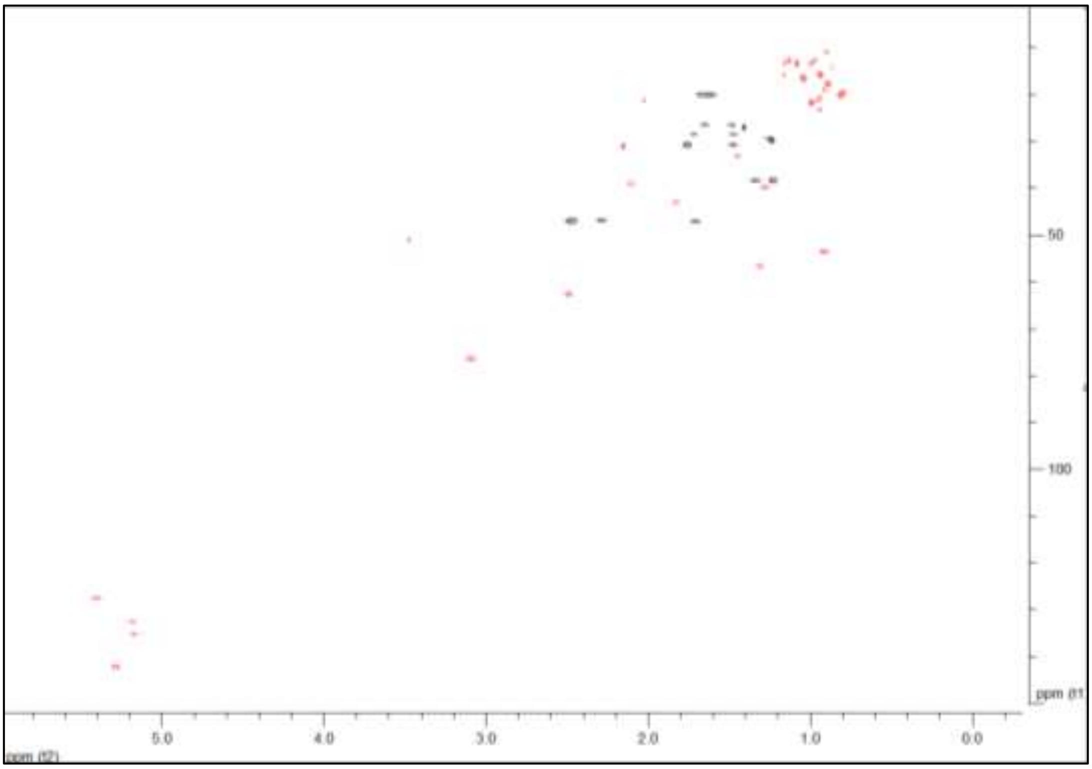

Figure S3. HSQC spectrum of (9*E*,22*E*,24*R*)-3*β*-hydroxy-4*α*,24-dimethyl-8,9-seco-5*α*-cholesta-9(11),22-dien-8-one (**1**) in $CDCl_3$.

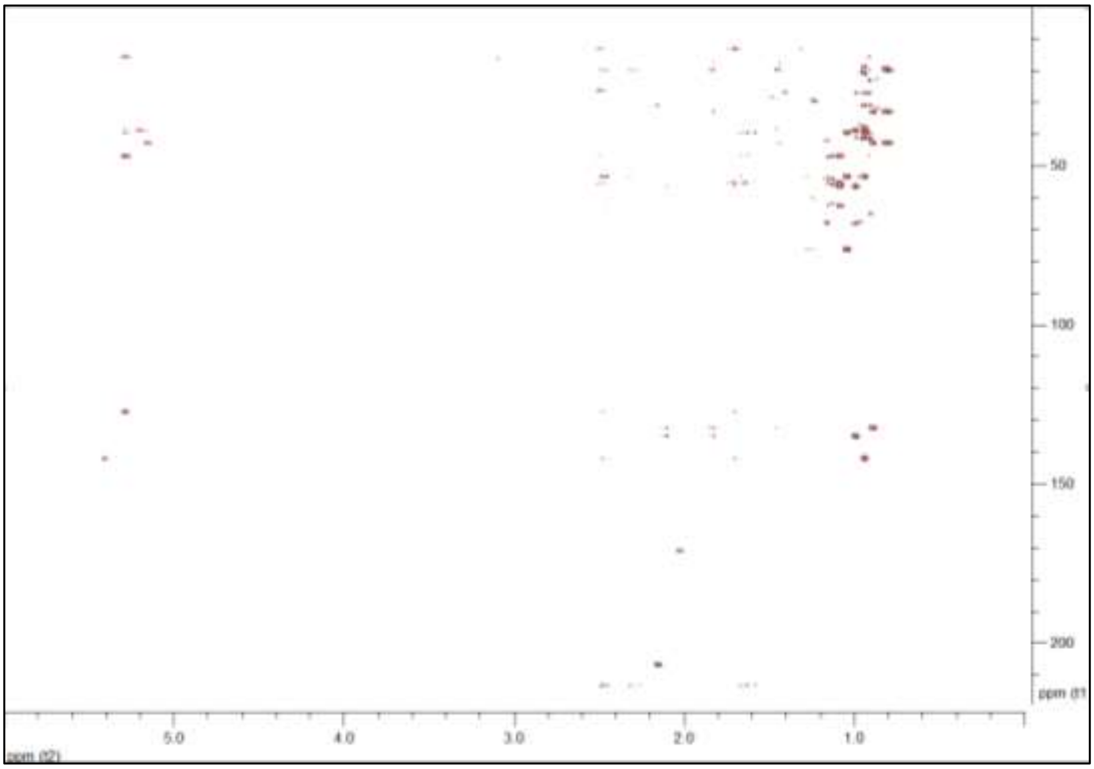

Figure S4. HMBC spectrum of (9*E*,22*E*,24*R*)-3*β*-hydroxy-4*α*,24-dimethyl-8,9-seco-5*α*-cholesta-9(11),22-dien-8-one (**1**) in $CDCl_3$.

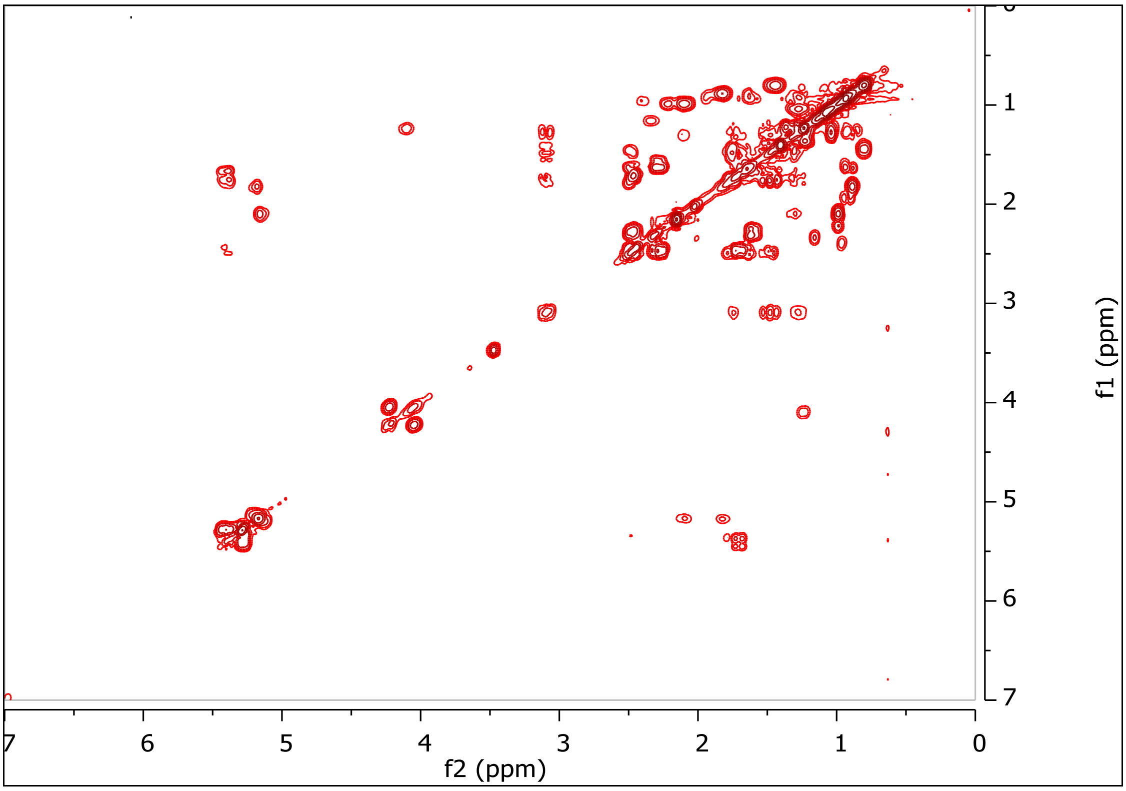

Figure S5. COSY spectrum of (9*E*,22*E*,24*R*)-3*β*-hydroxy-4*α*,24-dimethyl-8,9-seco-5*α*-cholesta-9(11),22-dien-8-one (**1**) in $CDCl_3$.

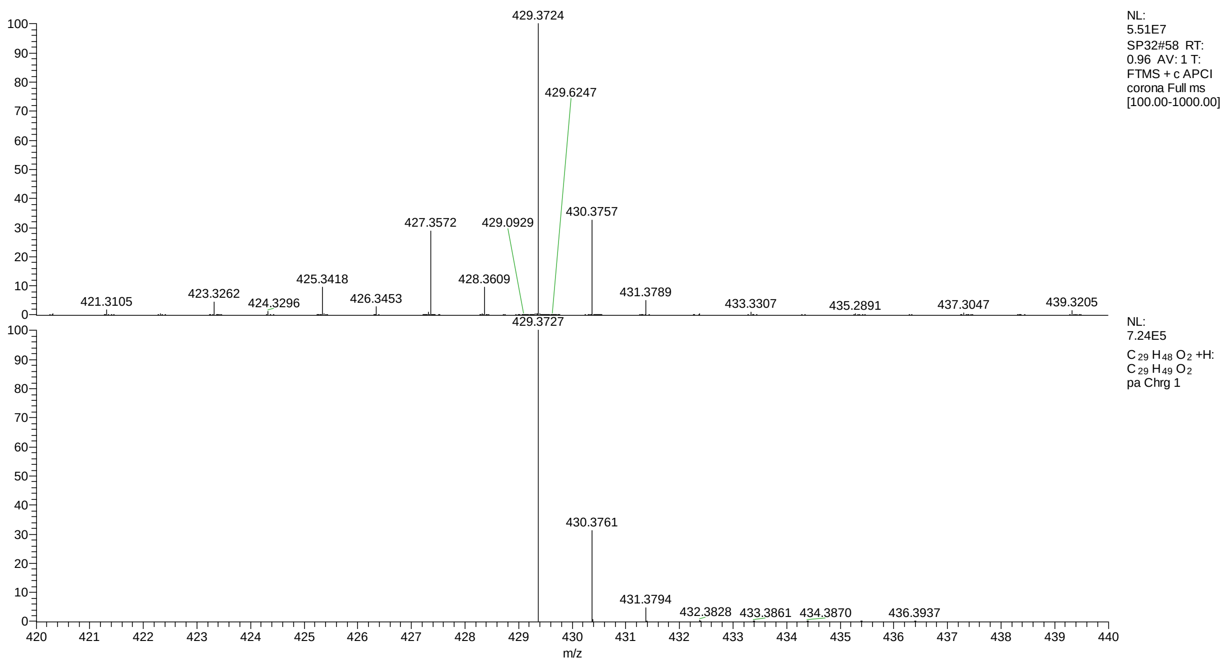

Figure S6. HR-APCIMS spectrum of (9*E*,22*E*,24*R*)-3*β*-hydroxy-4*α*,24-dimethyl-8,9-seco-5*α*-cholesta-9(11),22-dien-8-one (**1**).

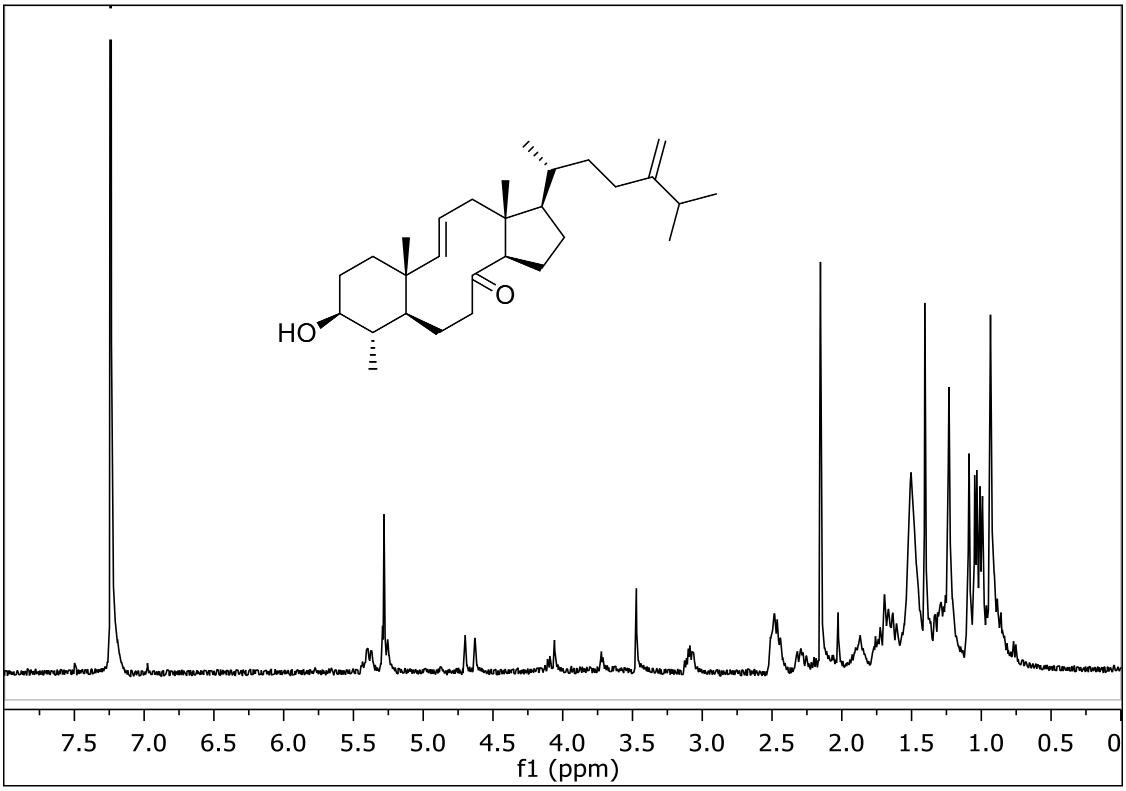

Figure S7. ^{1}H NMR spectrum of (9*E*)-3*β*-hydroxy-4*α*,24-dimethyl-8,9-seco-5*α*-cholesta-9(11),24(28)-dien-8-one (**2**) in $CDCl_3$.

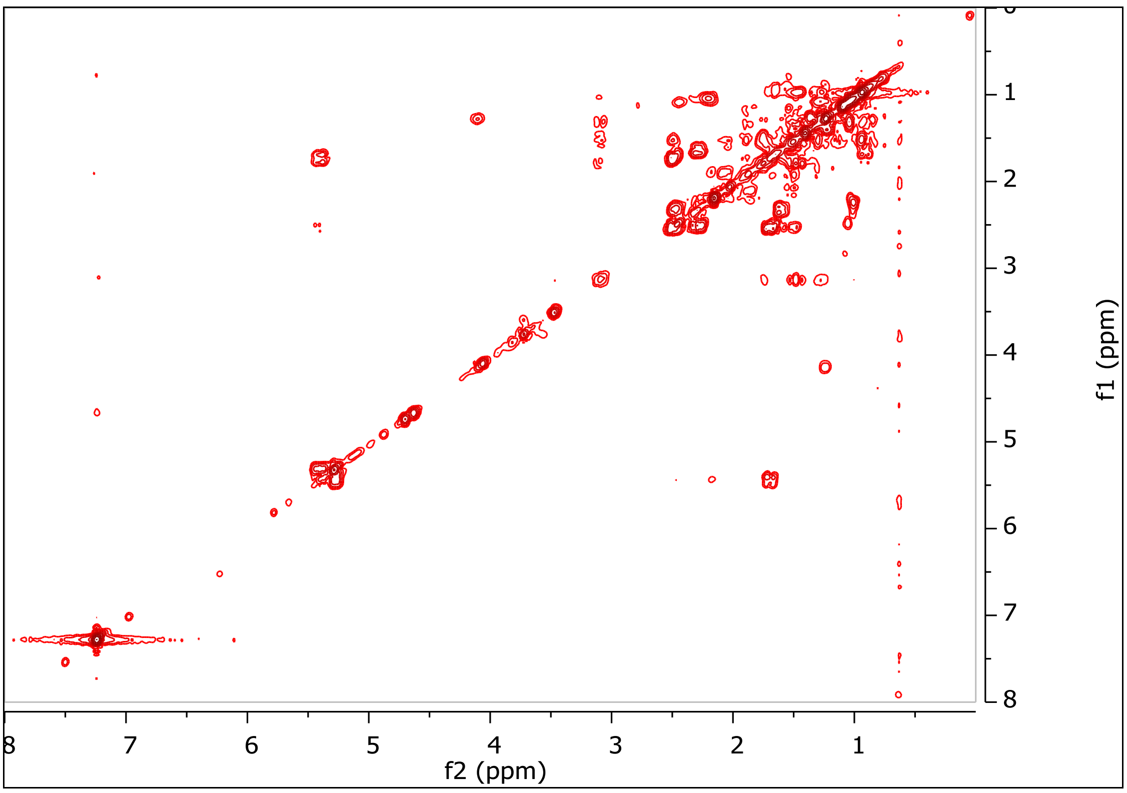

Figure S8. COSY spectrum of (9*E*)-3*β*-hydroxy-4*α*,24-dimethyl-8,9-seco-5*α*-cholesta-9(11),24(28)-dien-8-one (**2**) in $CDCl_3$.

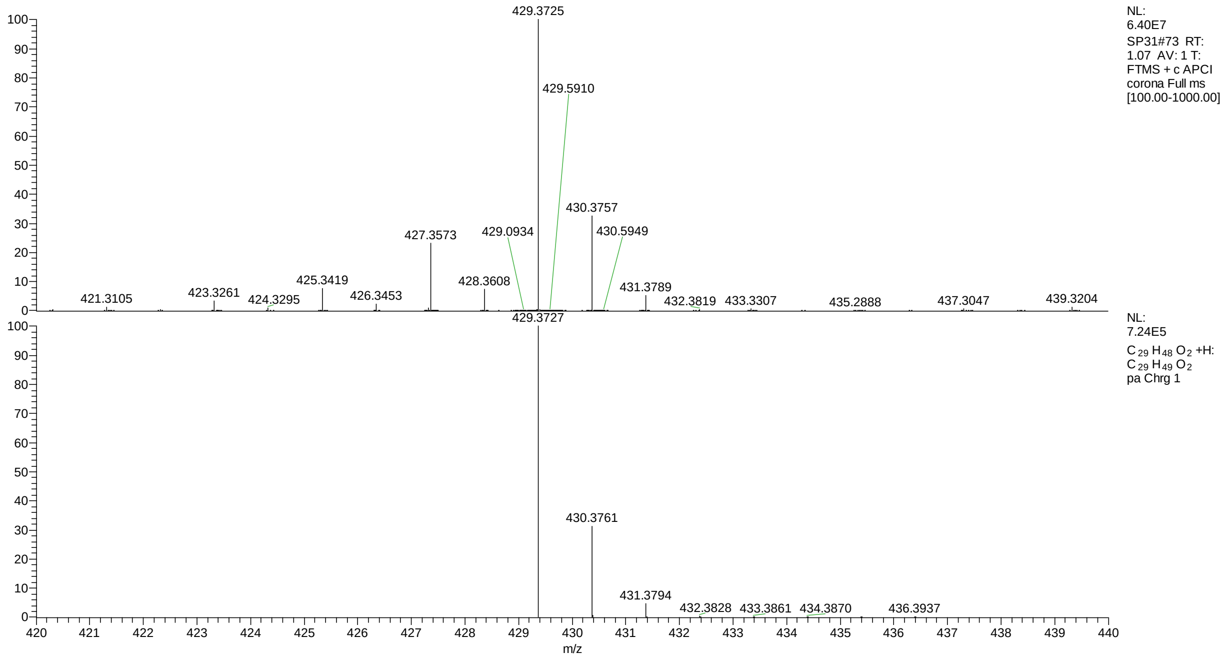

Figure S9. HR-APCIMS spectrum of (9*E*)-3*β*-hydroxy-4*α*,24-dimethyl-8,9-seco-5*α*-cholesta-9(11),24(28)-dien-8-one (**2**).

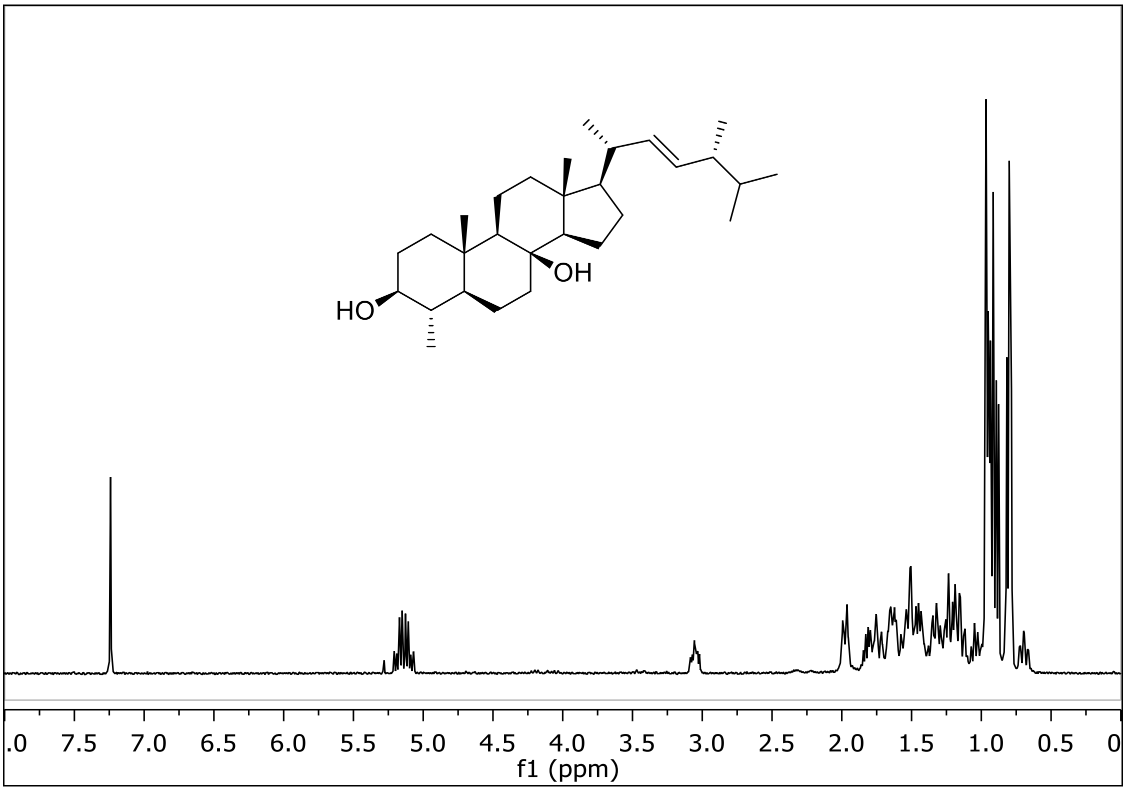

Figure S10. ^{1}H NMR spectrum of (22*E*,24*R*)-4*α*,24-dimethyl-5*α*-cholest-22-en-3*β*,8*β*-diol (**6**) in $CDCl_3$.

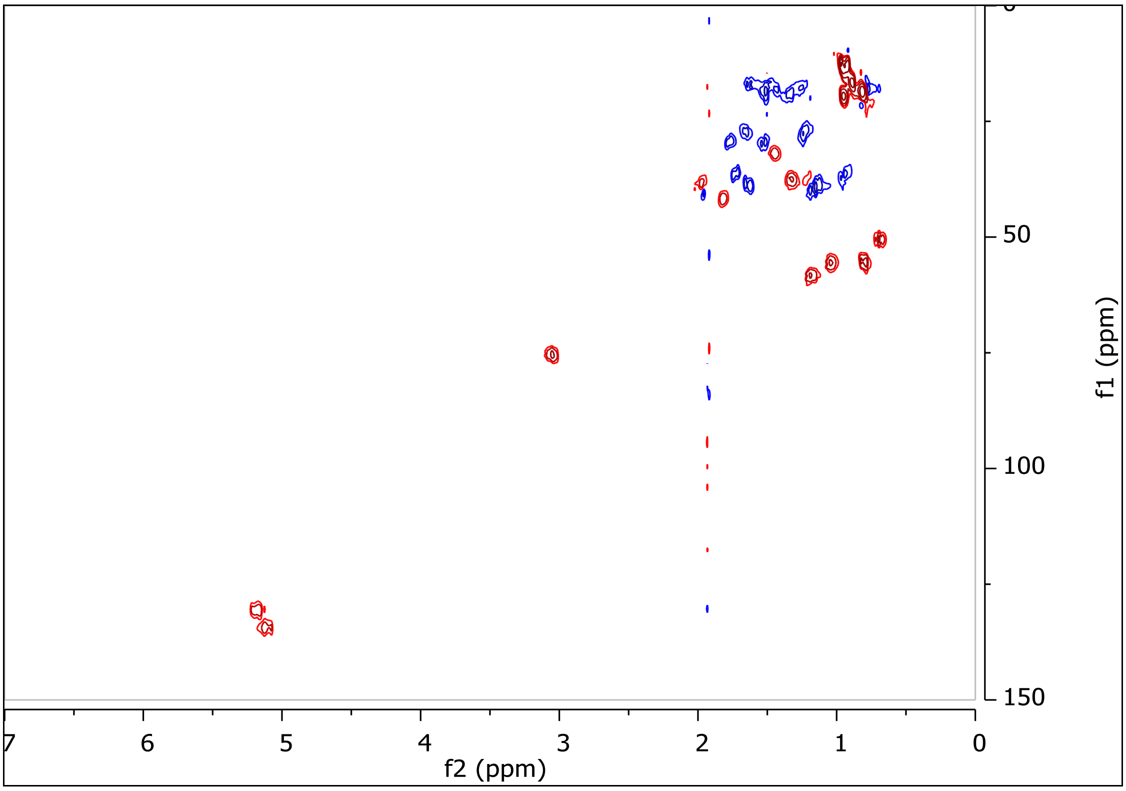

Figure S11. HSQC spectrum of (22*E*,24*R*)-4*α*,24-dimethyl-5*α*-cholest-22-en-3*β*,8*β*-diol (**6**) in $CDCl_3$.

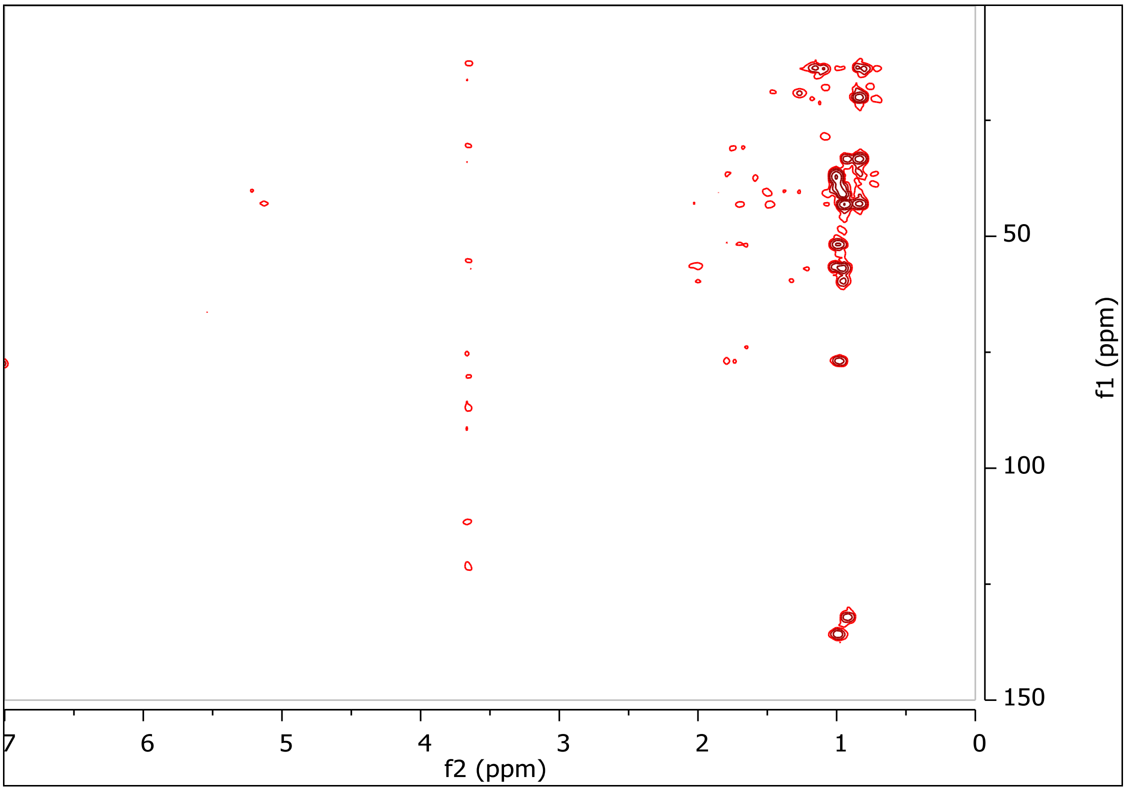

Figure S12. HMBC spectrum of (22*E*,24*R*)-4*α*,24-dimethyl-5*α*-cholest-22-en-3*β*,8*β*-diol (**6**) in $CDCl_3$.

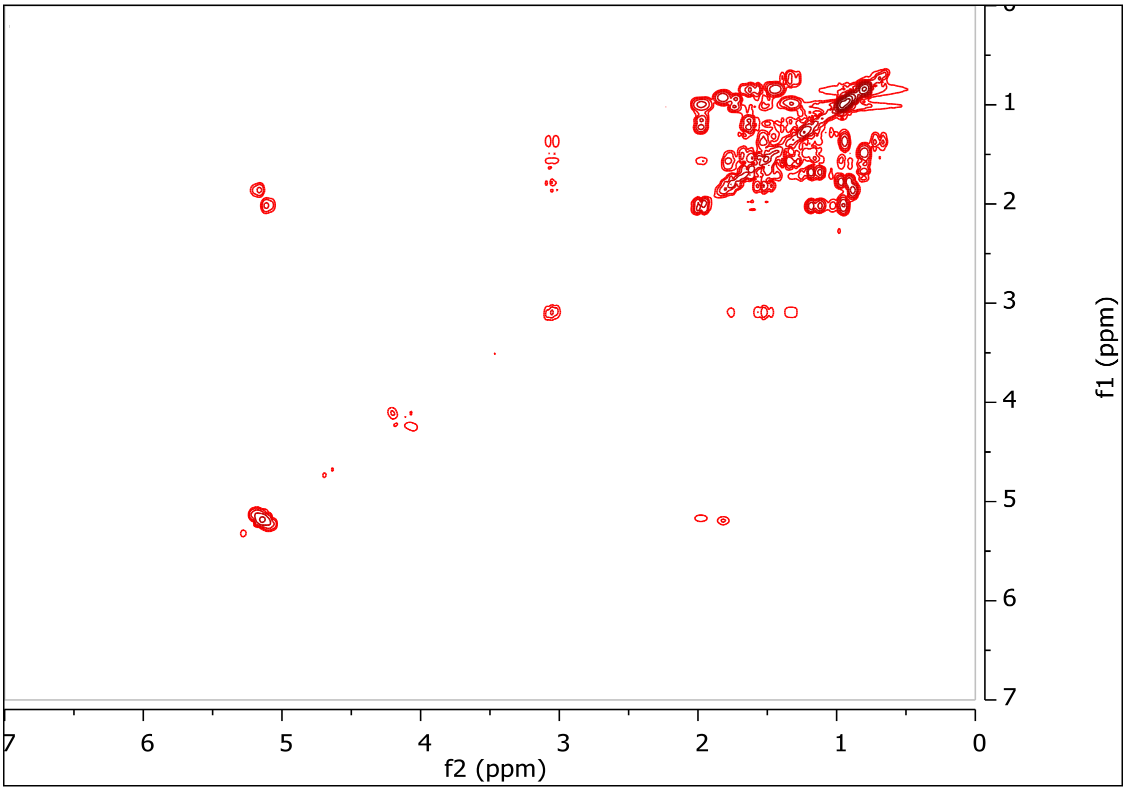

Figure S13. COSY spectrum of (22*E*,24*R*)-4*α*,24-dimethyl-5*α*-cholest-22-en-3*β*,8*β*-diol (**6**) in $CDCl_3$.

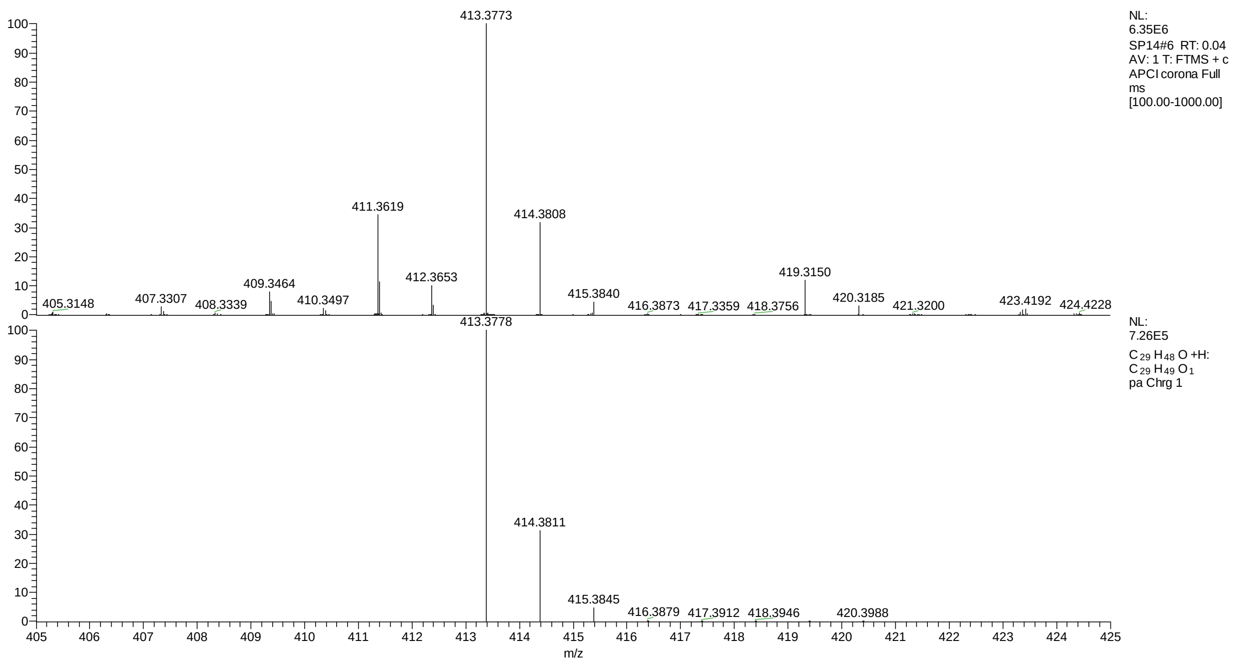

Figure S14. HR-APCIMS spectrum of (22*E*,24*R*)-4*α*,24-dimethyl-5*α*-cholest-22-en-3*β*,8*β*-diol (**6**).

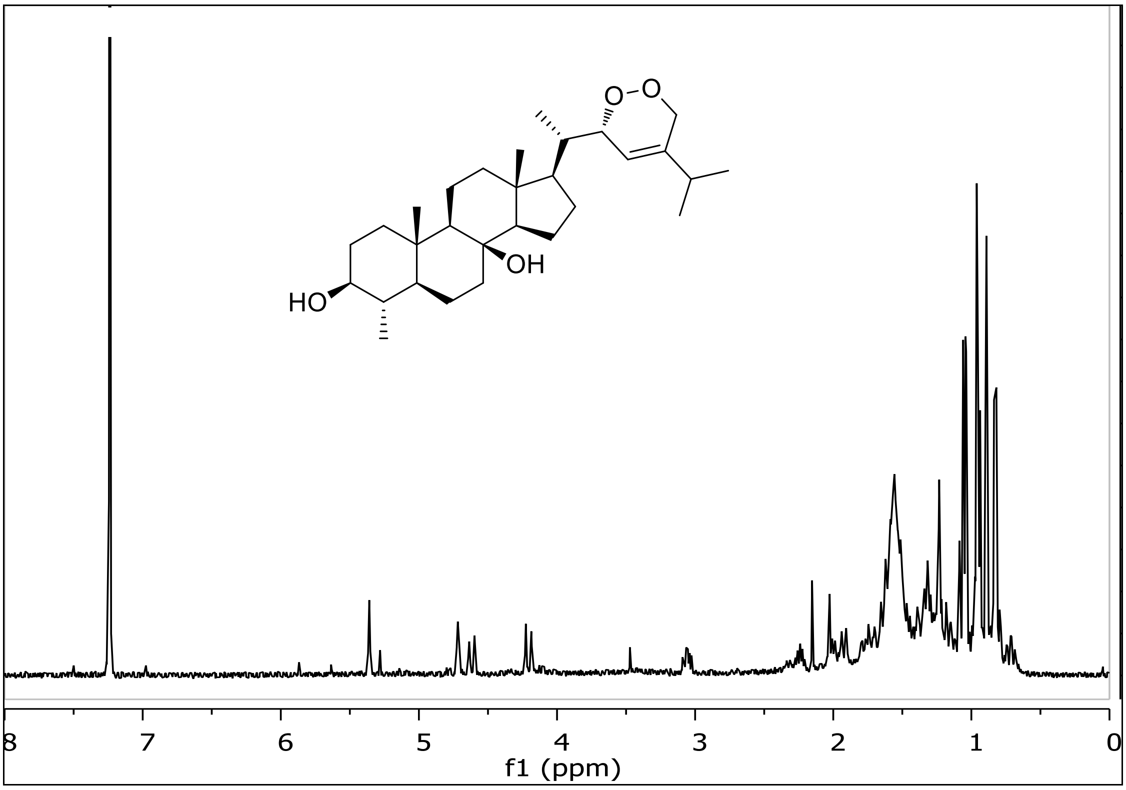

Figure S15. ^{1}H NMR spectrum of (23*E*)-22*α*,28-epidioxy-4*α*,24-dimethyl-5*α*-cholest-23-en-3*β*,8*β*-diol (**8**) in $CDCl_3$.

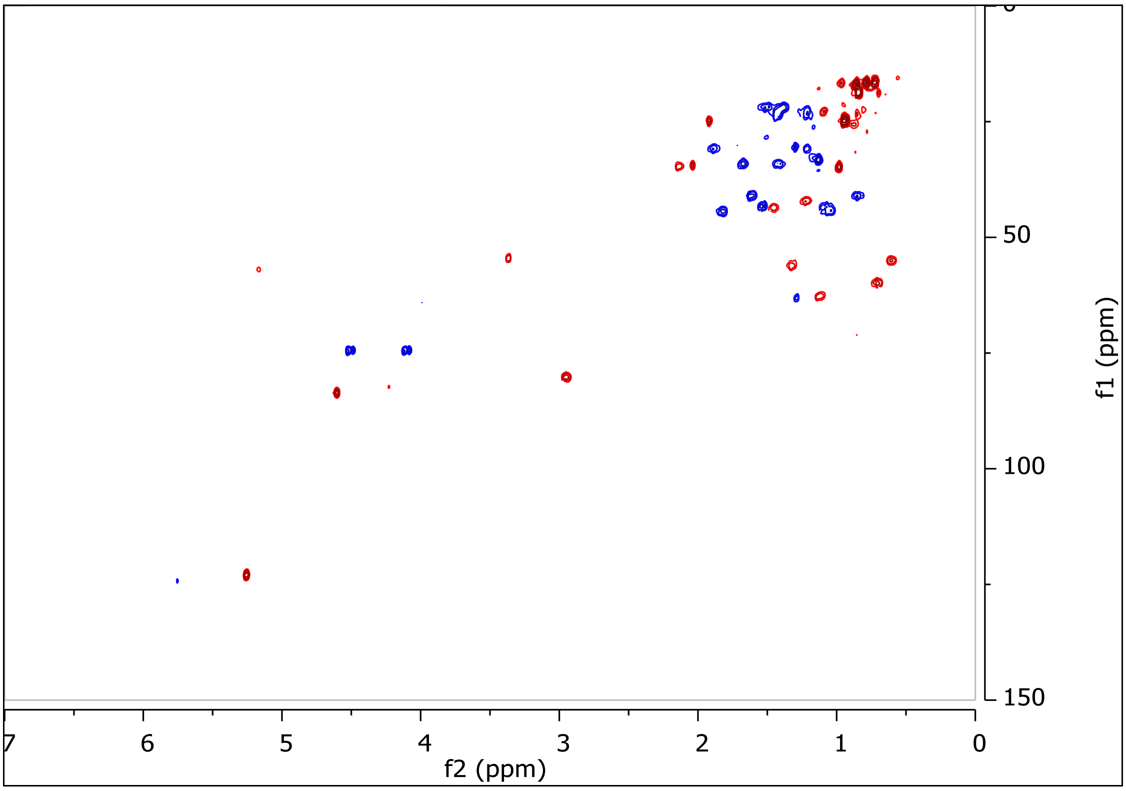

Figure S16. HSQC spectrum of (23*E*)-22*α*,28-epidioxy-4*α*,24-dimethyl-5*α*-cholest-23-en-3*β*,8*β*-diol (**8**) in CDCl3.

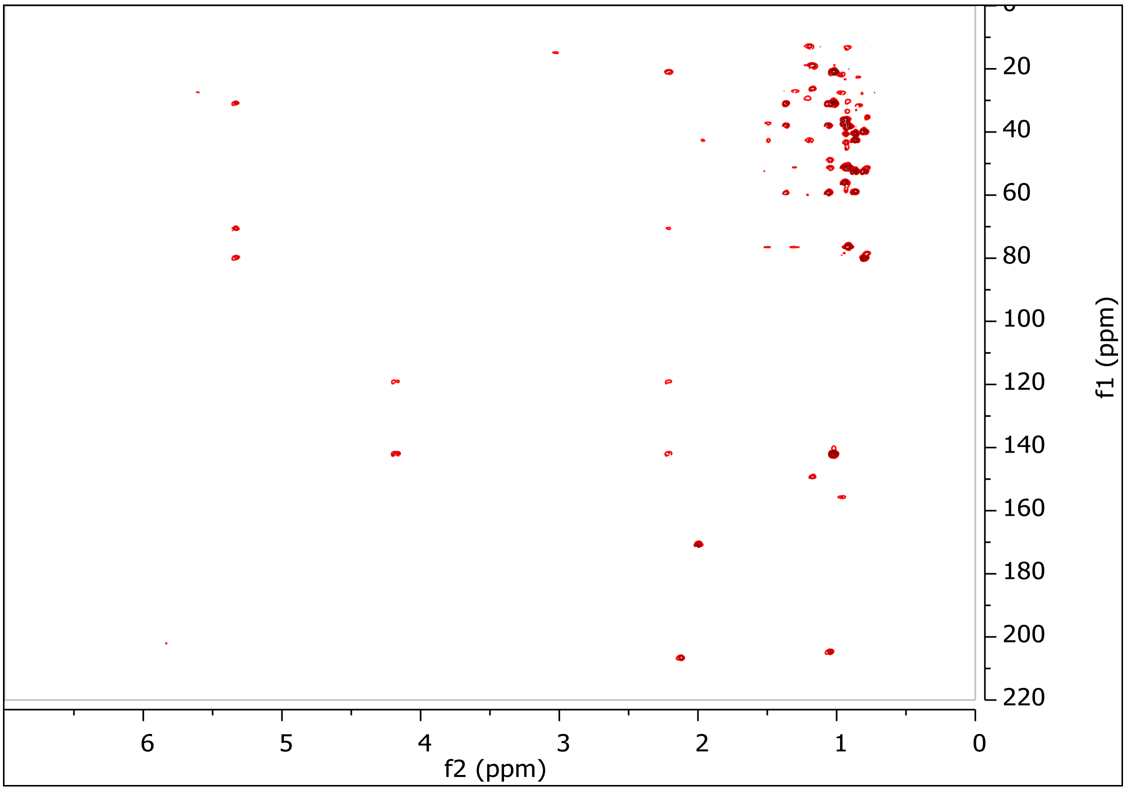

Figure S17. HMBC spectrum of (23*E*)-22*α*,28-epidioxy-4*α*,24-dimethyl-5*α*-cholest-23-en-3*β*,8*β*-diol (**8**) in CDCl3.

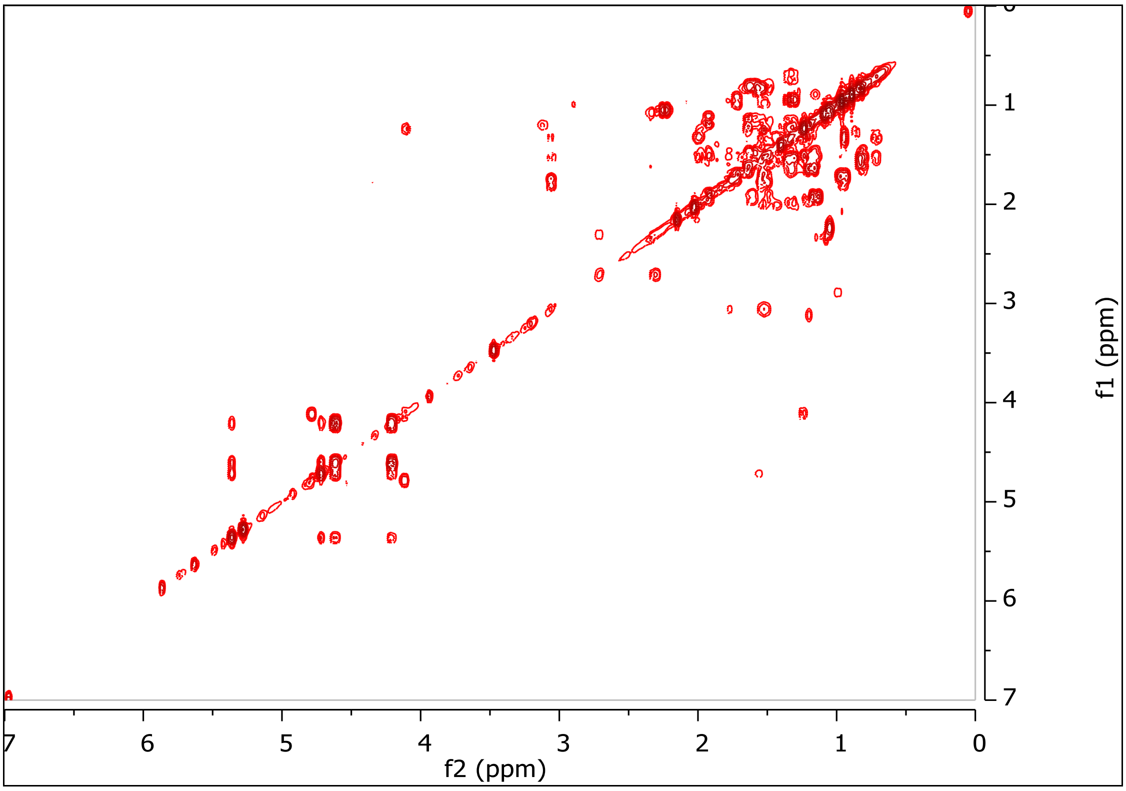

Figure S18. COSY spectrum of (23*E*)-22*α*,28-epidioxy-4*α*,24-dimethyl-5*α*-cholest-23-en-3*β*,8*β*-diol (**8**) in $CDCl_3$.

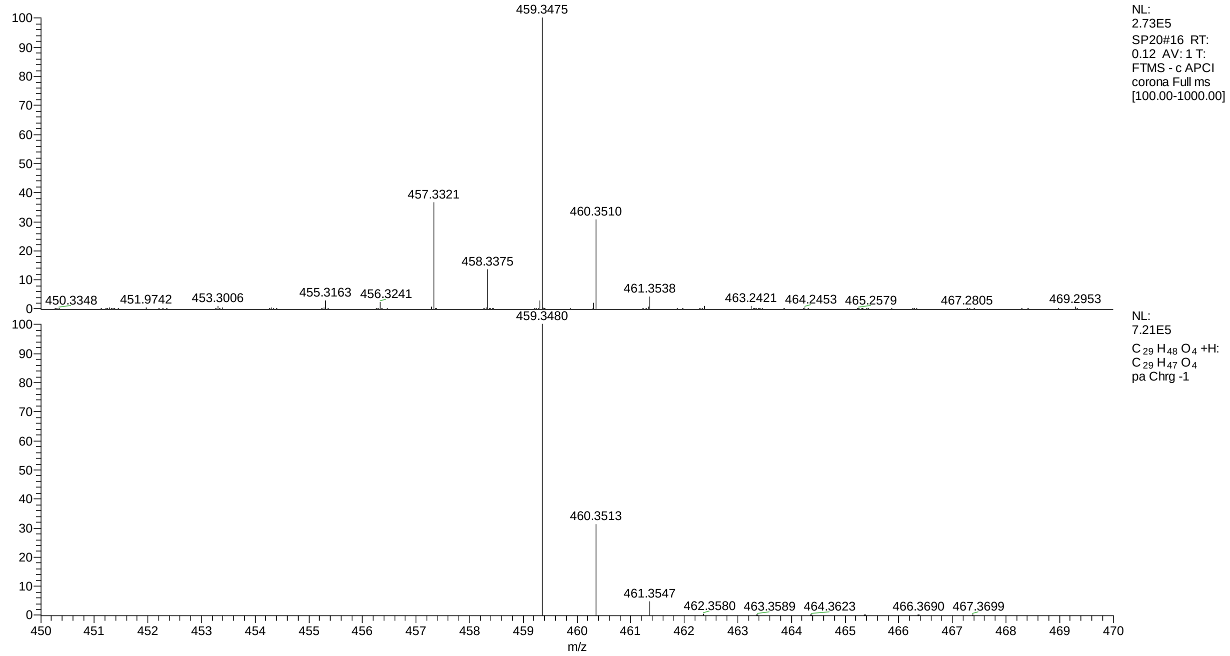

Figure S19. HR-APCIMS spectrum of (23*E*)-22*α*,28-epidioxy-4*α*,24-dimethyl-5*α*-cholest-23-en-3*β*,8*β*-diol (**8**).

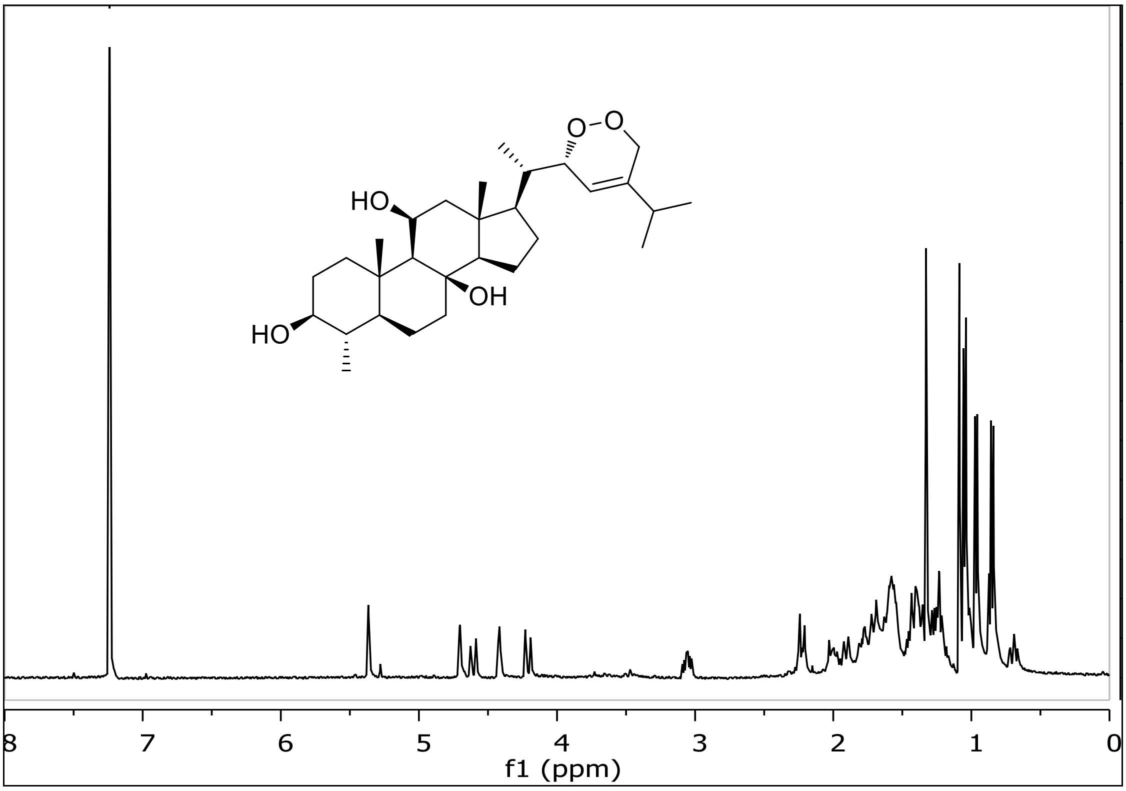

Figure S20. ^{1}H NMR spectrum of (23*E*)-22*α*,28-epidioxy-4*α*,24-dimethyl-5*α*-cholest-23-en-3*β*,8*β*,11*β*-triol (**13**) in $CDCl_3$.

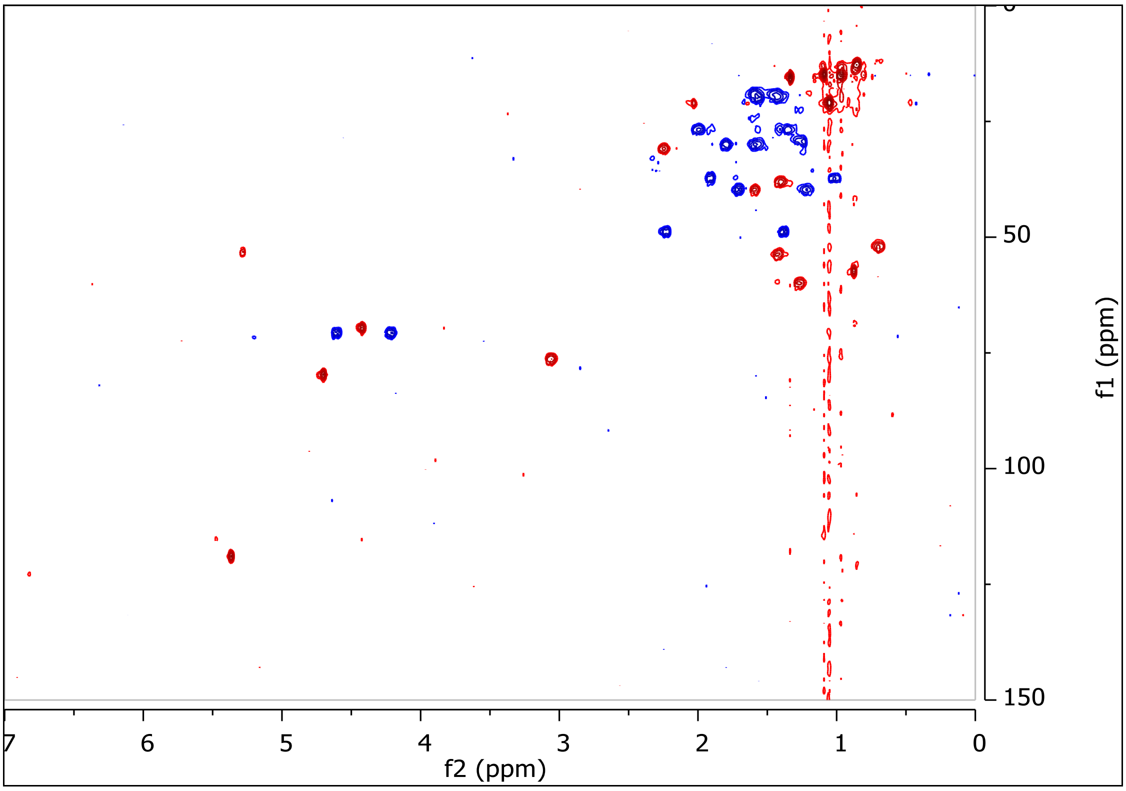

Figure S21. HSQC spectrum of (23*E*)-22*α*,28-epidioxy-4*α*,24-dimethyl-5*α*-cholest-23-en-3*β*,8*β*,11*β*-triol (**13**) in $CDCl_3$.

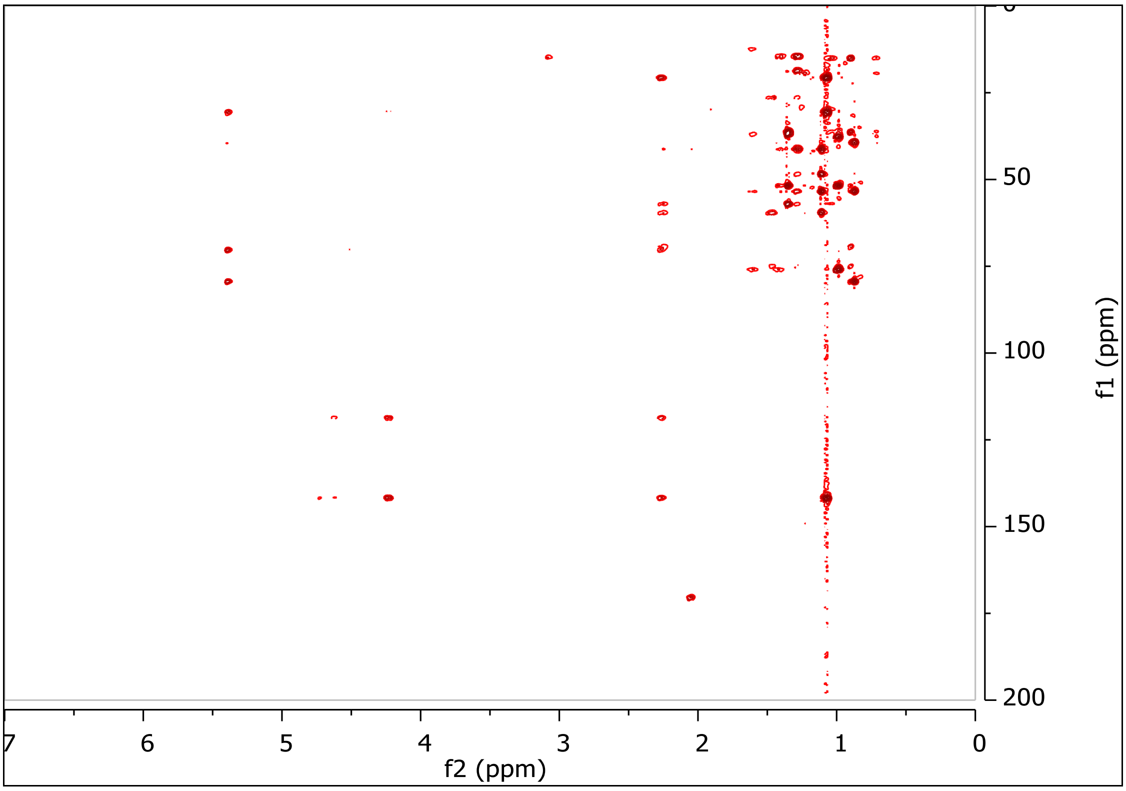

Figure S22. HMBC spectrum of (23*E*)-22*α*,28-epidioxy-4*α*,24-dimethyl-5*α*-cholest-23-en-3*β*,8*β*,11*β*-triol (**13**) in $CDCl_3$.

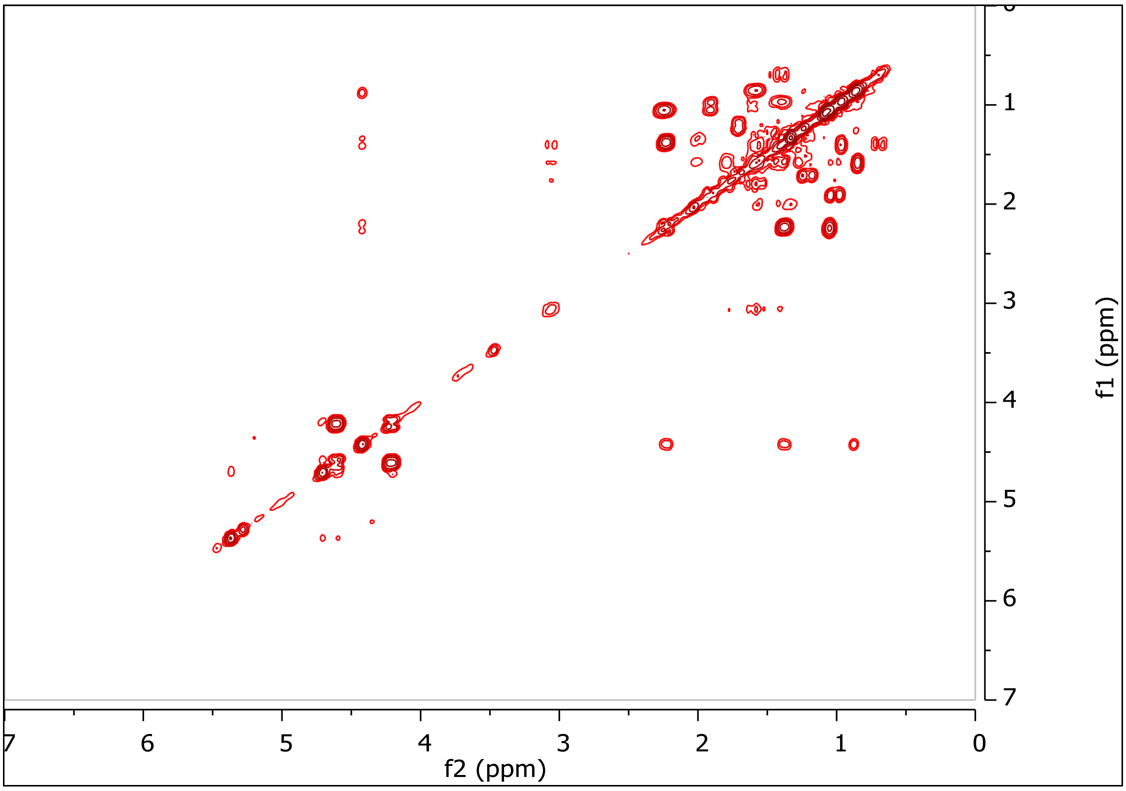

Figure S23. COSY spectrum of (23*E*)-22*α*,28-epidioxy-4*α*,24-dimethyl-5*α*-cholest-23-en-3*β*,8*β*,11*β*-triol (**13**) in $CDCl_3$.

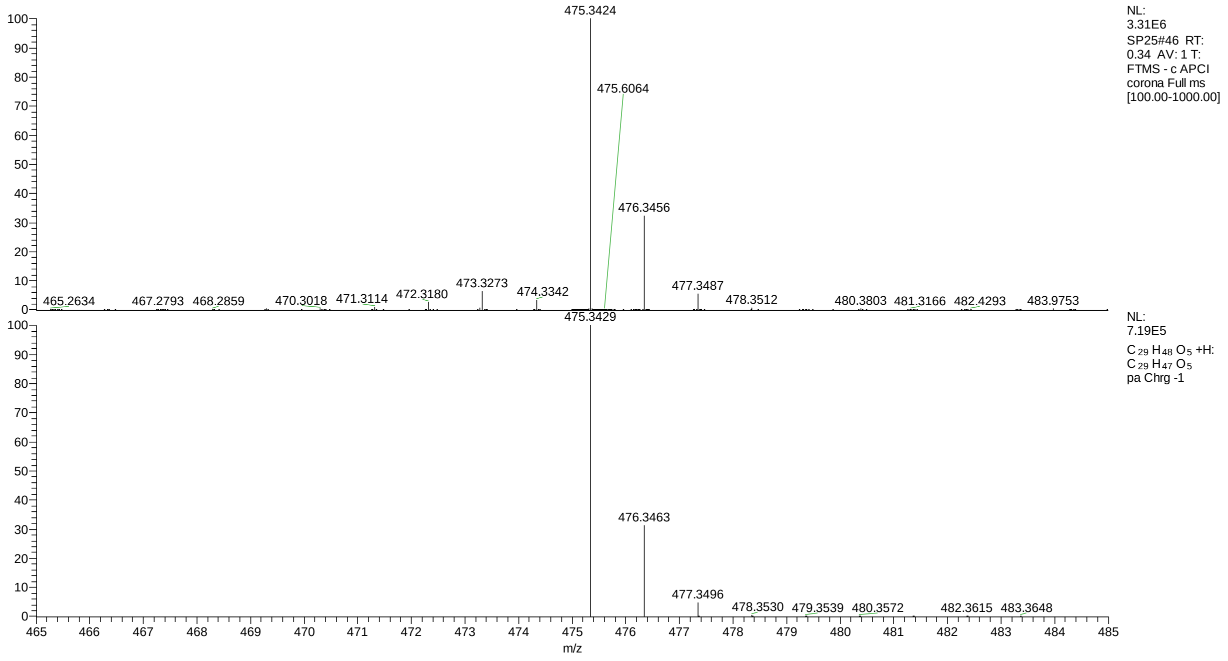

Figure S24. HR-APCIMS spectrum of (23*E*)-22*α*,28-epidioxy-4*α*,24-dimethyl-5*α*-cholest-23-en-3*β*,8*β*,11*β*-triol (**13**).

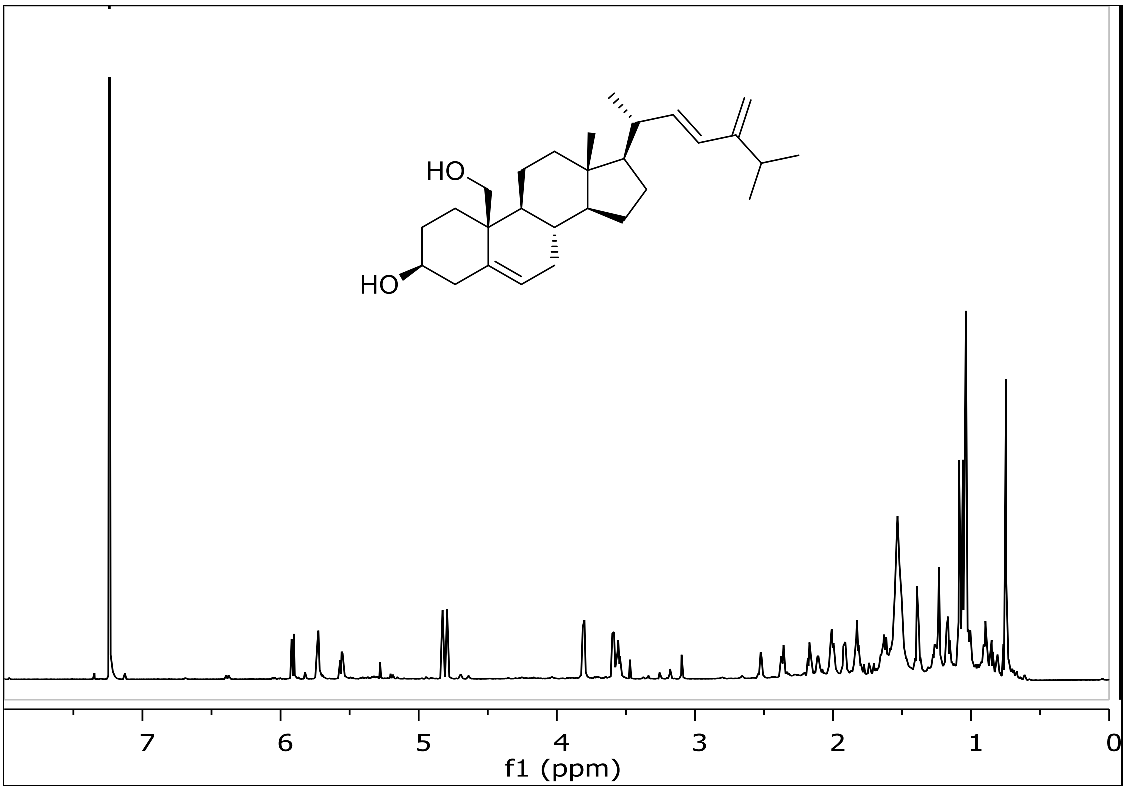

Figure S25. ^{1}H NMR spectrum of (22*E*)-24-methyl-cholesta-5,22,24(28)-trien-3*β*,19-diol (**20**) in $CDCl_3$.

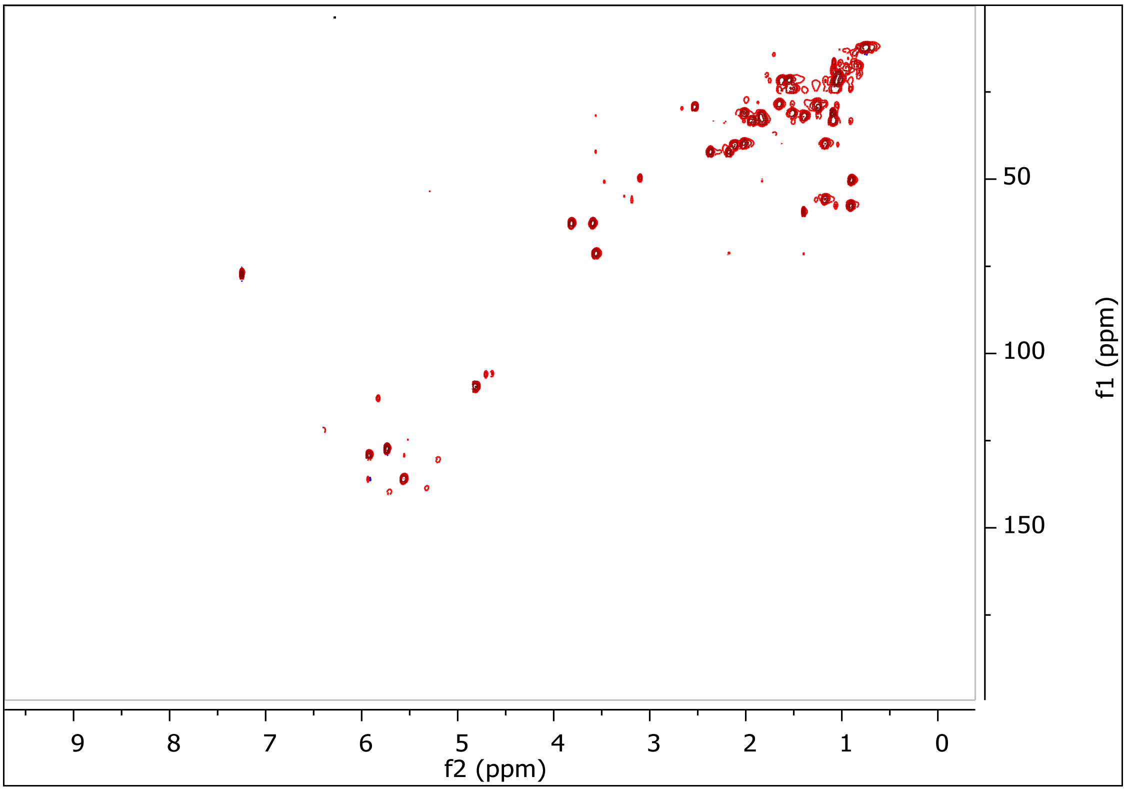

Figure S26. HSQC spectrum of (22*E*)-24-methyl-cholesta-5,22,24(28)-trien-3*β*,19-diol (**20**) in $CDCl_3$.

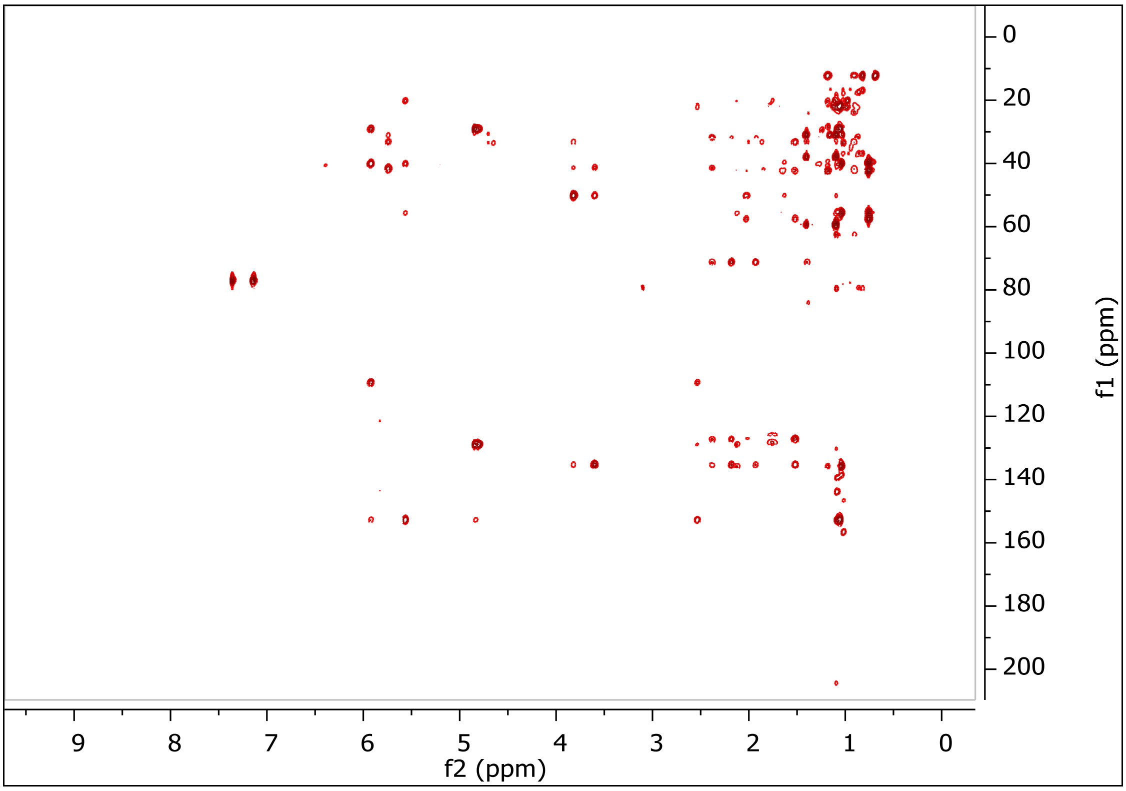

Figure S27. HMBC spectrum of (22*E*)-24-methyl-cholesta-5,22,24(28)-trien-3*β*,19-diol (**20**) in $CDCl_3$.

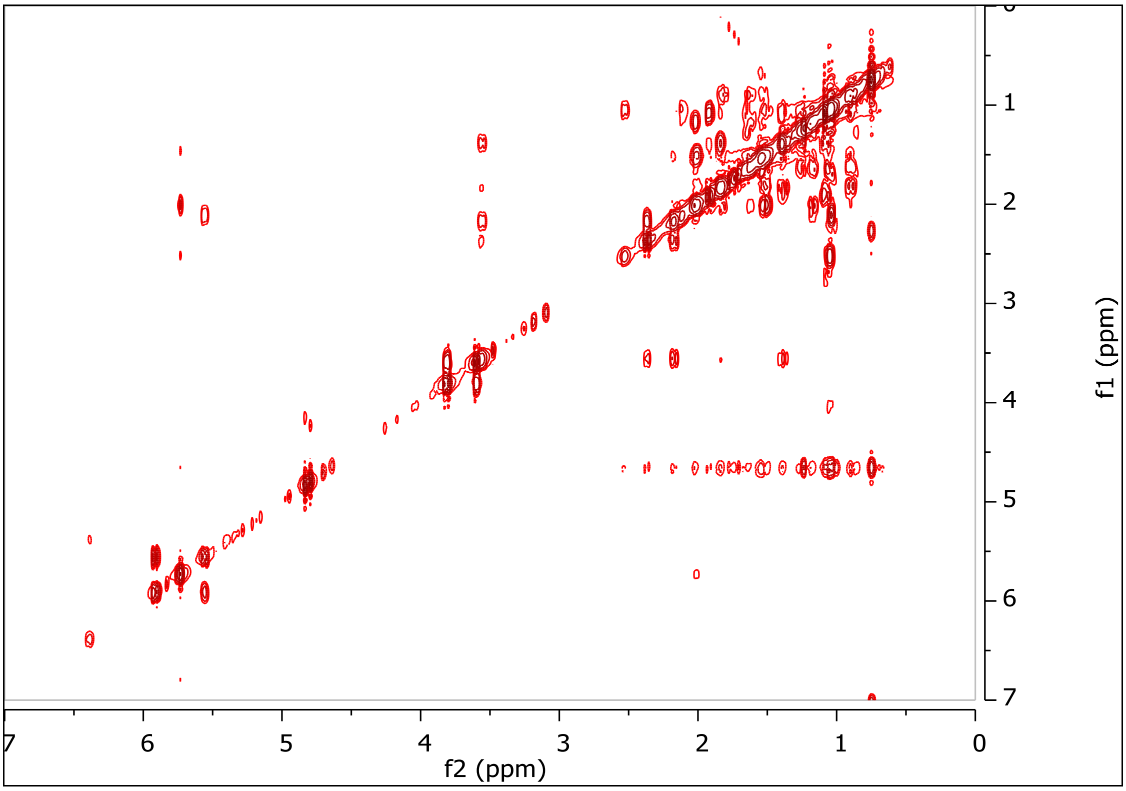

Figure S28. COSY spectrum of (22*E*)-24-methyl-cholesta-5,22,24(28)-trien-3*β*,19-diol (**20**) in $CDCl_3$.

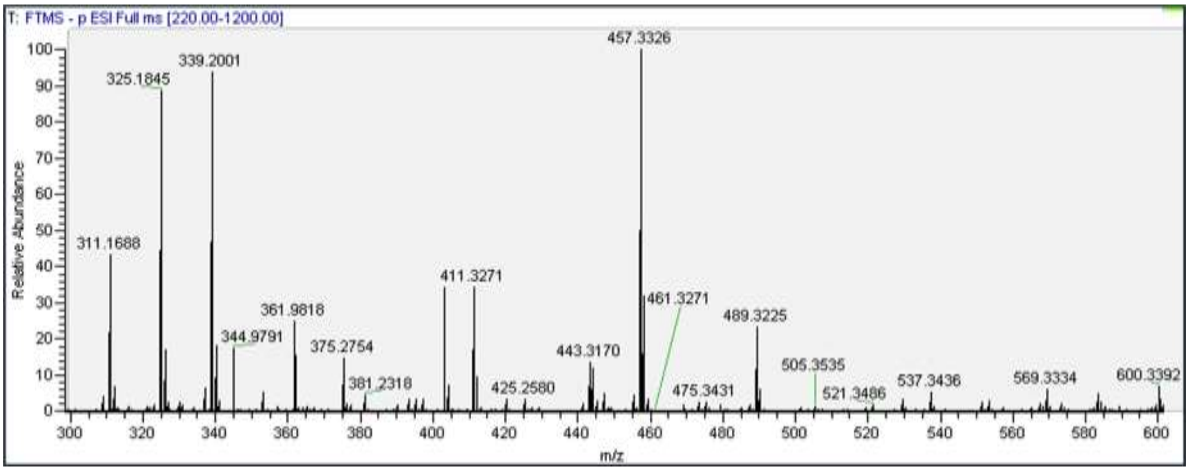

Figure S29. HR-ESIMS spectrum of (22*E*)-24-methyl-cholesta-5,22,24(28)-trien-3*β*,19-diol (**20**).
